# Sonochemically‐Induced Reduction of Alkenes to Alkanes with Ammonia

**DOI:** 10.1002/anie.202212719

**Published:** 2022-11-15

**Authors:** Anaelle Humblot, Tony Chave, Prince N. Amaniampong, Stéphane Streiff, François Jérôme

**Affiliations:** ^1^ Institut de Chimie des Milieux et Matériaux de Poitiers Université de Poitiers, CNRS 1 rue Marcel Doré, Bat B1 (ENSI-Poitiers) 86073 Poitiers France; ^2^ Univ Montpellier CNRS UMR 5257 ICSM, CEA, UM, ENSCM Marcoule France; ^3^ Eco-Efficient Products and Process Laboratory, SOLVAY/CNRS 3966 Jin Du Rd., Xin Zhuang Industrial Zone Shanghai 201108 China

**Keywords:** Alkenes, Ammonia, Hydrazine, Hydrogenation, Ultrasound

## Abstract

With the progressive defossilization of our industry, hydrogen (H_2_) has been identified as a central molecule to store renewable electricity. In this context, ammonia (NH_3_) is now rapidly emerging as a promising hydrogen carrier for the future. This game change indirectly impacts the field of fine chemistry where hydrogenation reactions are widely deployed. In particular, the possibility of performing hydrogenation reactions using ammonia directly instead of hydrogen has become highly desirable but it remains a very difficult scientific task, which we address in this communication. Here we show that the N−H bond of NH_3_ can be cleaved within cavitation bubbles, generated by ultrasonic irradiation at a high frequency, leading to the in situ formation of a diimide, which then induces the hydrogenation of alkenes. Advantageously, this work does not involve any transition metal and releases N_2_ as a sole co‐product.

## Graphical abstract

Ammonia is used as a hydrogen source for the reduction of alkenes to alkanes. The originality stems from the use of cavitation bubbles that act as “microreactors” to break the N−H bond of NH_3_. As a result, this protocol does not involve any catalyst and it releases nitrogen (and partly hydrogen) as a sole co‐product.

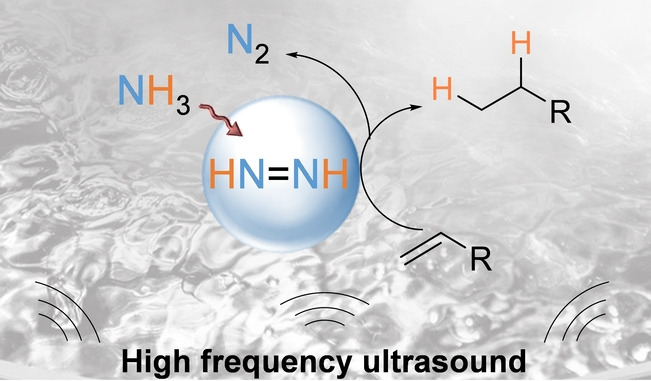

The massive defossilization of our society is currently leading to the rapid emergence of “green” hydrogen, in particular to store renewable electricity.[^1^, ^2^] In this context, ammonia is nowadays considered as the future hydrogen carrier.[^3^, ^4^] Developing hydrogenation reactions using directly ammonia (NH_3_) instead of hydrogen (H_2_) as a reductant is thus highly appealing but the high N−H bond dissociation energy of NH_3_ (435 kJ mol^−1^) represents an important hurdle faced by current catalytic technologies.

The hydrogenation of alkenes represents a very good example as it is one of the most widespread industrial reactions.^[5]^ The hydrogenation of alkenes is commonly catalyzed by transition metals in the presence of molecular hydrogen (Scheme 1).^[6]^ Although this reaction is very well optimized on a large scale, the use of pressure, the transportation and the storage of H_2_ imply drastic constraints that could be partly overcome by the direct utilization of NH_3_.[^3^, ^4^] Furthermore, with the increase interest of industry for reactions in water, the hydrogenation of alkenes faces limitations, mainly due to the very low solubility of H_2_ in water, an issue which is generally overcome by applying high pressures of H_2_ or by the design of specific equipment/catalysts to circumvent mass transfer problems.[^7^, ^8^]

**Scheme 1:**
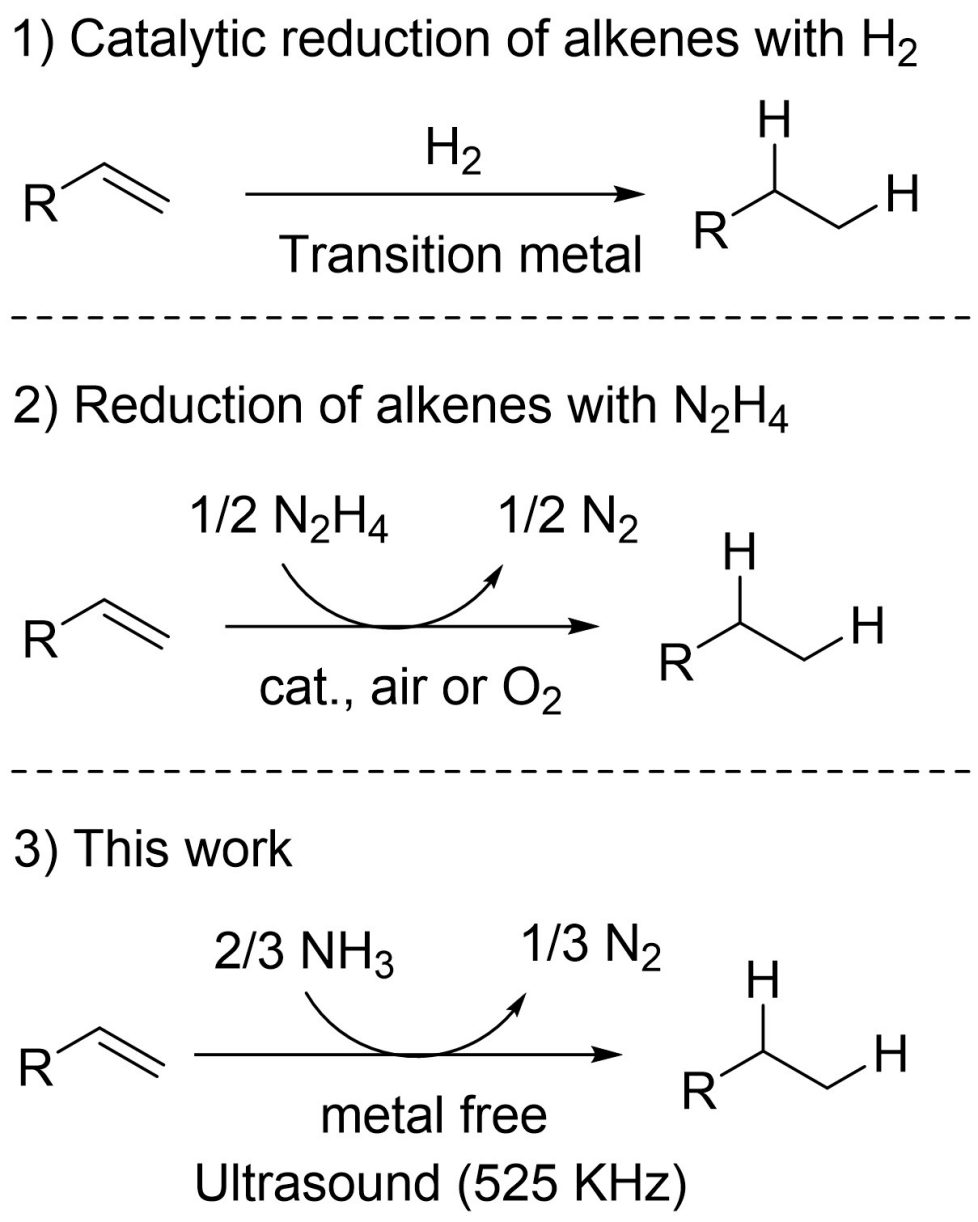
Positioning of the work in the state of the art.

The utilization of hydrazine (N_2_H_4_) as a reducing agent is now witnessing a sort of renaissance, in particular for aqueous phase hydrogenation reactions (Scheme 1).[^9^, ^10^] The mild reaction conditions and the release of safe N_2_ as a sole co‐product represent noticeable advantages. The key step is the in situ oxidation of N_2_H_4_ to diimide (HN=NH) which then readily reacts with alkenes yielding the corresponding alkanes and N_2_ (Figure S1). In situ oxidation of N_2_H_4_ to diimide is generally achieved by O_2_ (or H_2_O_2_),[^11^, ^12^, ^13^, ^14^] and this oxidation reaction can be accelerated by addition of a catalytic amount of transition metals (Cu,^[15]^ Pt,^[16]^ Mn,^[17]^ Fe,[^18^, ^19^, ^20^] Ni,^[21]^ Al^[22]^), either supported or not, or organocatalysts such as flavins.[^23^, ^24^, ^25^, ^26^] Even if the risk of detonation/flammability is very unlikely with hydrazine hydrate, at least below 69 wt % in water, the toxicity of aqueous hydrazine solutions represents the main drawback of this route, in particular for hydrazine concentrations higher than 6 wt %.[^27^, ^28^]

The possibility to produce in situ a diimide directly from NH_3_ instead of N_2_H_4_ is a very attractive option for hydrogenation reactions, but it remains a challenging task which we address in this work (Scheme 1). The decomposition of N_2_H_4_ to N_2_ and H_2_O (Δ*G*=−150 kJ mol^−1^) is indeed thermodynamically more favorable than the conversion of NH_3_ to N_2_H_4_ (Δ*G*=16.5 kJ mol^−1^). As a result, any catalyst capable of activating NH_3_ will unavoidably, and very quickly, decompose N_2_H_4_, making it nearly impossible the deployment of NH_3_‐based route for the catalytic reduction of alkenes.

Recently, we reported an alternative pathway to catalysis for the direct synthesis of N_2_H_4_ from NH_3_.^[28]^ In this previous work, NH_3_ was activated and converted to N_2_H_4_ by ultrasonic irradiation of aqueous NH_3_ solutions at a high frequency (525 kHz). This technology does not involve any catalyst, the N−H bond of NH_3_ was cleaved inside cavitation bubbles, where extreme conditions of pressure and temperature exist,^[29]^ leading to the formation of radical species (NH, ⋅NH_2_) which then recombined at the gas bubble‐liquid interface, forming N_2_H_4_. Advantageously, the bulk solution remained close to ambient temperature, thus limiting the thermal decomposition of N_2_H_4_ to N_2_ and H_2_O.

Inspired by this discovery, we report here the sonochemically‐induced hydrogenation of alkenes in water by using directly NH_3_ as a hydrogen source. So far, ammonia‐borane[^30^, ^31^, ^32^] and activation of NH_3_ by electrochemistry^[33]^ has been proposed for the hydrogenation of alkenes, but both processes rely on the use of transition metals, most of them being expensive and with limited availability and/or future risk of supply. To our knowledge, the catalyst‐free activation of cheap NH_3_ for hydrogenation of alkenes remains unsolved.

In a first set of experiments, we investigated the hydrogenation of 1‐octene in an aqueous solution of NH_3_ (5 wt %) subjected to an ultrasonic irradiation at 525 kHz. The temperature of the solution was maintained at 30 °C using a cooling jacket (Figure S2). 1‐octene was selected as a model substrate as it is a representative of cheap and non‐activated alkene widely processed on a large scale. Transposition to other alkenes is discussed later. As 1‐octene is not miscible in aqueous NH_3_ solution, it was deposited on the surface of an activated microporous carbon (DARCO®‐100) (5 wt % of 1‐octene) to facilitate its dispersion in water (Table S1). This strategy was also motivated by the principle of heterogeneous cavitation, i.e. the particles of carbon serve as nuclei for the formation and growth of cavitation bubbles, a mean to maximize the occurrence of cavitation events on the supported 1‐octene liquid phase.[^34^, ^35^]

Typically, 100 mg of activated carbon impregnated with 5 wt % of 1‐octene were suspended in 100 mL of aqueous NH_3_ (5 wt %) and sonicated at 525 kHz for the desired ultrasonic time. At the end of the ultrasonic irradiation, the carbon was recovered by filtration and washed with dichloromethane to desorb organic products. The dichloromethane phase was then analyzed by gas chromatography (GC) to determine the conversion, yield and selectivity (Figure S3–S6). The 1‐octene/*n*‐octane ratio was doubly checked by ^1^H NMR (Figure S7). Pleasingly, under these conditions, 12 % of 1‐octene was reduced to *n*‐octane within 2 h of ultrasonic irradiation, demonstrating the feasibility of this approach. Extending the ultrasonic time from 2 to 28 h led to a gradual increase of the *n*‐octane yield from 12 to 93 % (Scheme 2).

**Scheme 2:**
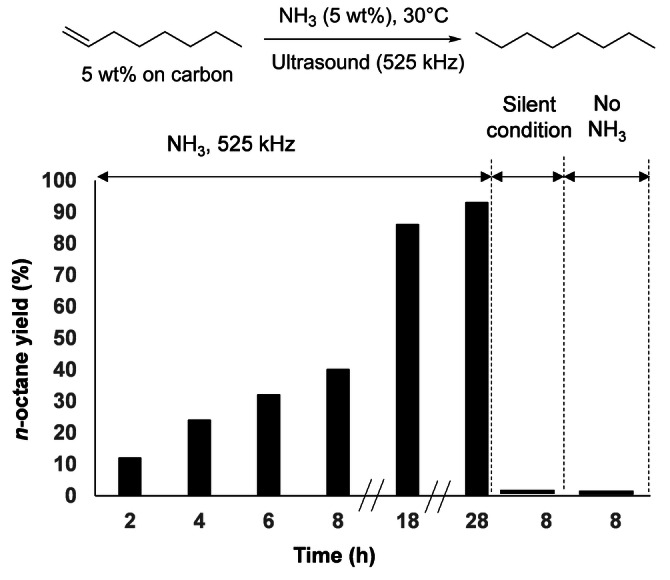
Kinetic profile and blank experiments (30 °C, 525 kHz, NH_3_ 5 wt %, under air).

No other product than *n*‐octane was detected by NMR, mass spectrometry (MS) and GC, revealing the high selectivity of this methodology. Under silent conditions (i.e. no ultrasonic irradiation) or in the absence of NH_3_, no *n*‐octane was formed and 1‐octene remained unaltered, confirming that the reduction of 1‐octene was a result of the combination of NH_3_ with high frequency ultrasound (Scheme 2). After removal of 1‐octene/*n*‐octane, the microporous carbon can be successfully reused at least three times without any noticeable impact on the *n*‐octane yield (Table S2). It is noteworthy that a slightly lower yield in *n*‐octane was obtained when the microporous carbon support was replaced by a mesoporous one (20 % *vs* 32 %), presumably because part of the entrapped 1‐octene is less exposed to cavitation events in the latter case (Table S1).

In agreement with our previous report,^[28]^ the reduction of 1‐octene was found optimal at a NH_3_ concentration of 5 wt %. Lower concentrations reduced the rate of the reaction while higher concentrations decreased the efficiency of cavitation effects (Figure S8). This results was linked to the decomposition of NH_3_ which is an endothermic reaction. As a result, when the concentration of NH_3_ within the cavitation bubbles is too high, the decomposition of NH_3_ rapidly consumes the energy of the cavitation bubbles, thus limiting the growth and fate of cavitation bubbles.^[28]^ Increasing or decreasing the amount of 1‐octene loaded on activated carbon impacted the yield in *n*‐octane measured after 6 h of ultrasonic irradiation. For instance, increasing the 1‐octene loading on carbon from 2 to 23 wt % linearly dropped the *n*‐octane yield from 36 to 10 % (Figure S9). However, in all cases, yields in *n*‐octane can be increased by extending the ultrasonic irradiation time over 6 h. This result suggests that the rate of the reaction is controlled by the ultrasonic activation of NH_3_. Furthermore, addition of radical scavengers such as phenol or sodium benzoate considerably inhibited the reaction suggesting the occurrence of a radical mechanism (Figure S10). Finally, the possible contribution of the metallic reactor wall (Inox 316 L) on the reaction mechanism has been ruled out by compartmenting the solution in a polyethylene flask (more information on SI, Figure S11)

At this stage, two hypothesis can be drawn for the radical reduction of 1‐octene (1) a complete decomposition of NH_3_ to N_2_ and H_2_, this latter can be further activated inside cavitation bubbles (formation of radical ⋅H) to reduce 1‐octene (additional amount of H_2_ may also stem from the sonolysis of H_2_O) or (2) the in situ formation of a diimide species (NH=NH) which is involved in the N_2_H_4_‐mediated reductive process. To discriminate between these two hypotheses, hydrogen was bubbled in a suspension of 1‐octene supported over activated carbon in water and subjected to an ultrasonic irradiation at 525 kHz (Scheme 3, top)

**Scheme 3:**
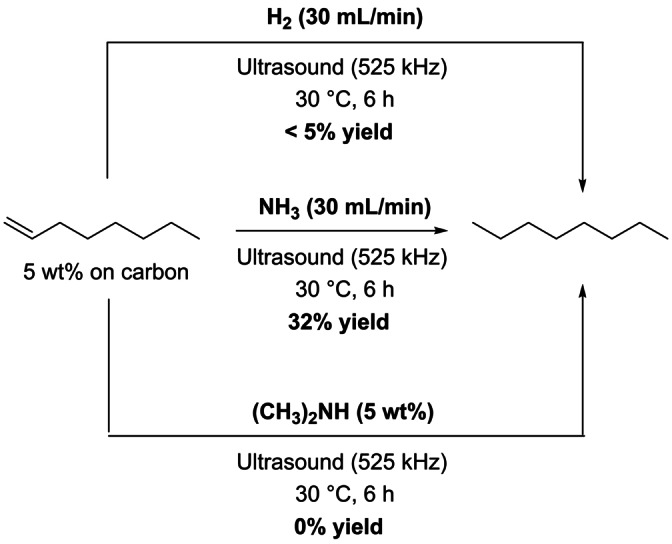
Control experiments with H_2_ and dimethylamine ((CH_3_)_2_NH).

Negligible formation of *n*‐octane was observed, indicating that a reduction with in situ produced H_2_ is less likely to occur (Scheme 3, Table 1, entry 2). This result can be rationalized by the very low solubility of H_2_ in water which prevents its activation by cavitation bubbles, as discussed in previous reports on sonochemistry.^[36]^ Hence, a reduction of 1‐octene by diimide appears to be the most plausible pathway. The key in situ formation of a diimide was further supported by replacing the aqueous solution of NH_3_ by an aqueous solution of dimethylamine (5 wt %); in this case a diimide can no longer be formed. As anticipated, in this case, no reaction occurred (Scheme 3, bottom).

**Table 1:** Impact of ultrasound, gas atmosphere and composition of the solution on the *n*‐octane yield.^[a]^

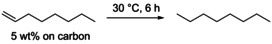				
Entry	Ultrasound (525 kHz)	Solution composition	Atmosphere	*n*‐octane yield [%]
1	Yes	NH_3_ (5 wt %)	Air	32
2	Yes	H_2_O	H_2_	<5%
3	Yes	NH_3_ (5 wt %)	Ar	46
4	Yes	H_2_O	N_2_	0
5^[b]^	No	NH_3_ (5 wt %)+1.25 mM N_2_H_4_	Air	22
6^[b]^	No	NH_3_ (5 wt %)+1.25 mM N_2_H_4_	Ar	<5%
7	Yes	NH_3_ (5 wt %)+0.7 mM N_2_H_4_	Ar	50
8	Yes	NH_3_ (5 wt %)+1.3 mM N_2_H_4_	Ar	52

[a] 525 kHz 6 h, 100 mg of carbon coated with 5 wt % of 1‐octene, 30 °C; [b] under silent conditions, results were collected after 2 h.

In our conditions, diimide can be formed according to two different scenarios (1) by direct recombination of biradical NH species, previously detected by sonoluminescence of aqueous NH_3_ solution^[37]^ or (2) by oxidation of in situ produced N_2_H_4_ either by dissolved air,[^11^, ^12^, ^13^, ^14^] ⋅OH radicals stemming from the sonolysis of water (also detected by sonoluminescence)^[37]^ or by the carbon itself.^[38]^ To clarify this point, ultrasonic experiments were first conducted under argon to assess the role of dissolved air. After 6 h of ultrasonic irradiation under argon, the yield to *n*‐octane was even higher (46 %), suggesting that dissolved air plays no major role in the formation of diimide under ultrasonic irradiation (Table 1, entry 3). Note that the improvement of sonochemical reaction rates under argon is due to the monoatomic nature of this gas which exhibit a high polytropic index and a low thermal conductivity, a pivotal aspect to optimize cavitation events.^[39]^ This experiment under argon also eliminates the possible formation of NH species by recombination of N_2_ from the air with H_2_O in the cavitation bubbles, as observed elsewhere.^[40]^ This was further confirmed by the absence of reaction when NH_3_ was replaced by a bubbling of N_2_ (Table 1, entry 4). Hence, to discriminate between the role of ⋅OH radicals from that of the carbon support, the reactivity of 1‐octene supported over activated carbon with N_2_H_4_ was investigated under silent conditions (*i.e* without ultrasound). As expected, at 30 °C under air, 1‐octene was reduced to *n*‐octane with 22 % yield in the presence of N_2_H_4_ (1.25 mM) (Table 1, entry 5). However, when air was replaced by an Ar atmosphere, the reduction of 1‐octene was nearly completely inhibited indicating that, during ultrasonic irradiation under Ar, the carbon support has no major role in the oxidation of N_2_H_4_ (Table 1, entry 6). This claim was further supported by FT‐IR analysis of the surface of activated carbon before and after ultrasonic irradiation which did not reveal any noticeable change in the surface composition (Figure S12). Altogether, our results strongly suggest that diimide is formed either by direct recombination of NH biradical generated within the cavitation bubbles or by oxidation of in situ produced N_2_H_4_ by ⋅OH radicals stemming from the sonolysis of water.

To get more information on the reaction mechanism, additional experiments were conducted. First, the amount of free hydrazine formed into the solution was titrated by spectrophotometry (Figure S13). Formation of hydrazine was observed, at a rate of 0.017 mmol/h, which is about 8 times lower than in the absence 1‐octene supported on carbon^[28]^ confirming that activated nitrogen species mainly reacted on the carbon surface. On the other hand, while ⋅OH species were previously evidenced by sonoluminescence of aqueous NH_3_ solution,^[37]^ no H_2_O_2_ was detected in our case which may be rationalized by its rapid in situ consumption, for instance by reaction with hydrazine to form the diimide.[^11^, ^12^, ^13^, ^14^] Next, the ultrasonic‐assisted reduction of 1‐octene was performed with incremental addition of N_2_H_4_ at the beginning of the reaction, and under Ar to prevent the formation of diimide by oxidation of N_2_H_4_ with air. Results were all collected after 6 h of ultrasonic irradiation. Initial addition of 0.7 and 1.3 mmol L^−1^ of N_2_H_4_ (i.e. 1.5 and 3.0 equiv relative to 1‐octene, respectively) did not significantly impact the yield in *n*‐octane after 6 h of reaction, showing that hydrazine is not the limiting reactant (Table 1, entries 3, 7, 8). A plot of the hydrazine concentration as a function of the ultrasonic time showed that hydrazine accumulated in the reactor at a nearly similar rate, whatever the amount of hydrazine initially introduced (Figure S14). Altogether, these experiments strongly supports that the rate of the hydrogenation reaction is limited by the formation rate of NH and ⋅OH species.

From all these results, one may propose a plausible reaction mechanism (Scheme 4). During ultrasonic irradiation, cavitation bubbles are formed on the surface of carbon particles, grow and then implode towards the surface, thus propelling the activated species (mainly NH and ⋅OH) on the 1‐octene layer. Two pathways are then possible for the generation of the diimide (1) a direct recombination of NH species or (2) the formation of hydrazine and then oxidation to diimide with ⋅OH radicals. These two routes probably co‐exist but, at this stage, it was difficult for us to accurately discriminate which route was the dominant one. Once the diimide is formed, it reacts with supported 1‐octene to yield *n*‐octane and N_2_. It should be noted that no trace of *n*‐octylamine was detected (by NMR, GC and MS) indicating that ⋅NH_2_ radicals, possibly formed by cracking of NH_3_ inside the cavitation bubbles, reaction of NH with ⋅H radicals, NH with NH_3_ or ⋅OH with NH_3_, very rapidly recombine at the gas bubble‐liquid interface to form hydrazine (Scheme 4). Finally, ⋅H radical can also recombine to form H_2_ which readily escape from the reaction due to its very low solubility in water.^[36]^

**Scheme 4:**
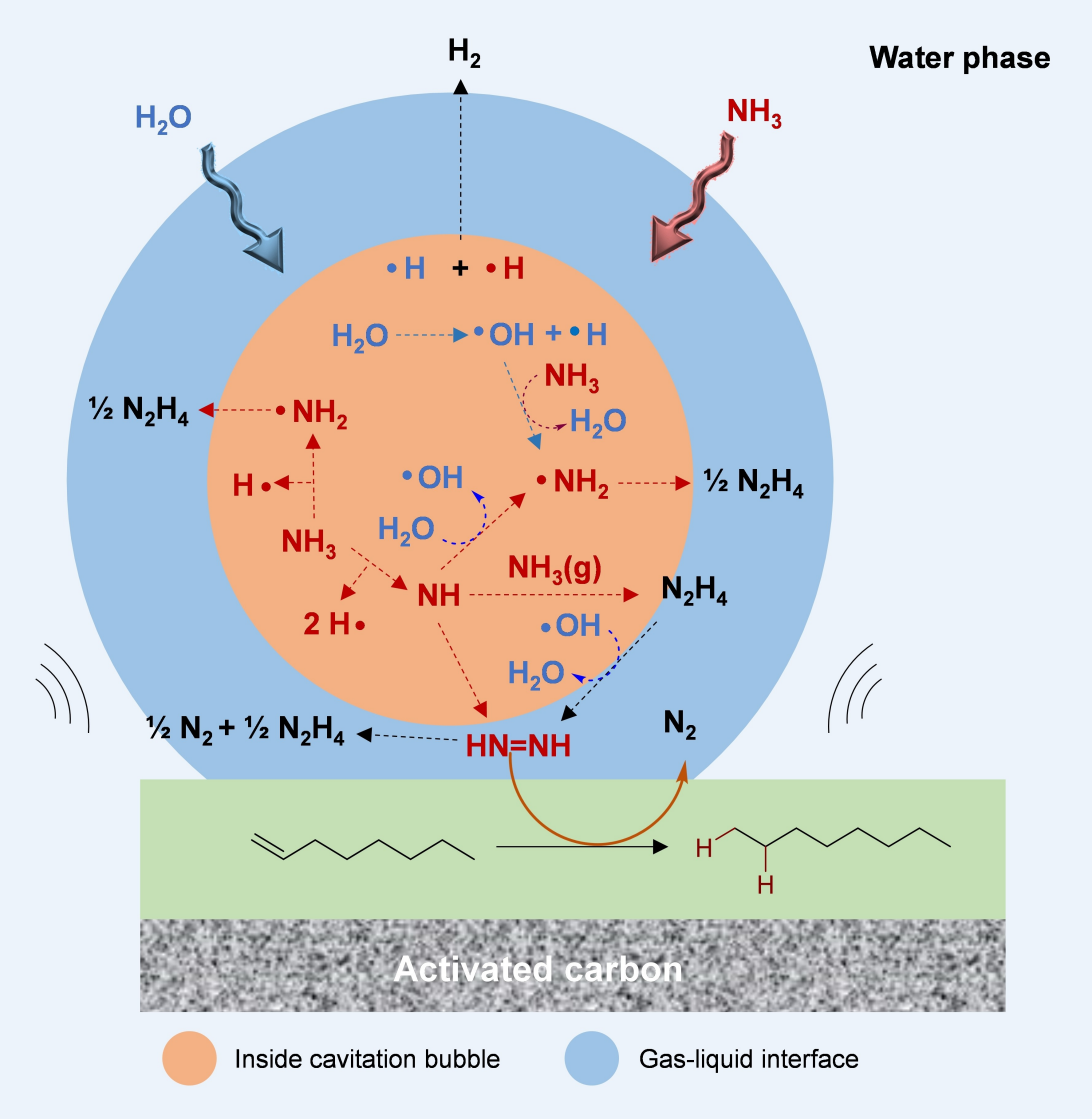
Proposed reaction mechanism illustrating the implosion of aqueous NH_3_ filled cavitation bubbles on the surface of activated carbon coated with 1‐octene

Assuming this proposed mechanism, the recombination of NH or ⋅NH_2_ with ⋅OH radicals, forming nitrites, nitrates and NOx, cannot be ruled out and may represent a limitation. To this end, the formation of nitrites and nitrates was monitored as a function of the ultrasonic reaction time (Figure S15). To our delight, in contrast to air, no nitrites/nitrates (at least below the detection limit (<0.5 mg mL^−1^)) were detected under argon (Figure S15), indicating that the recombination of NH or ⋅NH_2_ with ⋅OH radical is less likely to occur under argon. It also indirectly provides an experimental evidence that the amount of water (source of ⋅OH radical) in the cavitation bubble is probably much lower than that of NH_3_, a logical result considering that NH_3_ is a gas.

This concept was then applied to other relevant alkenes to demonstrate its potential (Table 2). Using linear alkenes such as 1‐dodecene and 1‐heptene, the corresponding alkanes were obtained with 45–65 % yield after 6 h of ultrasonic irradiation (Table 2, entries 1, 2). Using more activated alkenes such as cyclooctene or norbornene obviously led to a higher conversion; cyclooctane and norbornane were formed with 77 and 71 % yield, respectively, after 6 h (Table 2, entries 3, 4). From 1,7‐octadiene, a mixture of 1‐octene (29 %) and *n*‐octane (40 %) was obtained (Table 2, entry 5). Starting from 2‐octene, *n*‐octane was formed with 17 % yield, indicating that internal C=C bonds are also reactive under our reaction conditions (Table 2, entry 6), albeit with a lower reactivity than terminal C=C bonds. In this context, methyl oleate, an industrially relevant biobased alkene, was successfully reduced with this methodology, affording methyl stearate with 18 % yield (Table 2, entry 7). Alkyne such as 1‐octyne was also reduced affording, after 6 h, a mixture 1‐octene (26 %) and *n*‐octane (29 %) (Table 2, entry 8). In all cases, yields to the corresponding alkanes can be improved by extending the reaction time over 6 h.

**Table 2:** Transposition to various alkenes and alkyne.^[a]^

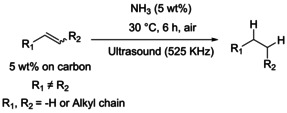		
Entry	Reactant	Product [% yield]
1	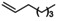	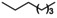
		65 %
		
2	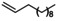	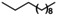
		45 %
		
3	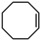	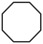
		77 %
		
4	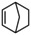	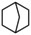
		71 %
		
5	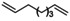	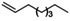
		29 %
		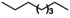
		40 %
		
6	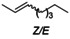	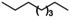
		17 %
		
7	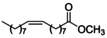	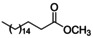
		18 %
		
8	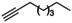	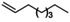
		26 %
		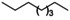
		29 %

[a] 5 wt % of alkene or alkyne supported on 50 mg of activated carbon, 525 kHz, 30 °C, 6 h, under air.

The predicted massive electrification of our society now opens opportunities for the research on new technologies such as ultrasounds but also on others like plasma, electrolysis, photocatalysis, γ‐radiation, etc. Although renewable electricity at a competitive price is now available from wind or solar,^[41]^ the amount of formed reactive species with these technologies (radicals in the case of high frequency ultrasound) is generally low, thus leading to low reactor productivities. Although it is too early at this stage to discuss about scale‐up, we noticed that the rate of sonochemically‐induced reactions can be drastically improved by increasing the acoustic power density (*i.e* W per mL). To do so, the volume of the solution was decreased from 150 to 50 mL (for a same energy input of 21 W), resulting in a concomitant increase of the acoustic power density from 0.14 to 0.42 W mL^−1^. This increase of the acoustic power density led to an improvement of the *n*‐octane yield (collected after 6 h of ultrasonic irradiation) from 11 to 68 %, respectively (Figure S16). Decreasing again the volume of the solution below 50 mL was technically not possible with our reactor configuration (due to overheating), but these last results demonstrate that the rate of sonochemically‐induced reduction reaction with NH_3_ could be theoretically and drastically improved by switching from a batch to a continuous flow reactor. Furthermore, in terms of energy saving, it is also noteworthy that free hydrazine formed into the solution could be advantageously used to speed up the hydrogenation reaction. Indeed, there is no need to push the sonochemically‐induced hydrogenation of alkene to complete conversion, as the in situ produced hydrazine could be further used to terminate the hydrogenation reaction under silent conditions (under air) as shown in Table 1 (entry 5). For instance, if the ultrasonic irradiation of the aqueous NH_3_ solution (5 wt %) was stopped after 25–30 % conversion of 1‐octene, there would be enough hydrazine into the solution to terminate, in theory, the hydrogenation of 1‐octene using catalysts such as flavins (Figure S17).[^23^, ^24^, ^25^, ^26^] Beside optimization of the ultrasonic reactor, this shows that the coupling of ultrasound (for the activation and conversion of NH_3_) to current catalytic processes (for the N_2_H_4_‐mediated hydrogenation of 1‐octene) is also a scientific line of thought to advantageously reduce the overall energy expenditure of this technology.

In conclusion, this work opens a path for the direct utilization of NH_3_, the future hydrogen carrier, instead of H_2_ for the reduction of alkenes to alkanes. This challenging reaction was tackled in this work by applying an ultrasonic irradiation, at a high frequency, in an aqueous solution of NH_3_. The as‐formed cavitation bubbles act as microreactors for the cracking of NH_3_ and the generation of a diimide which subsequently induces the hydrogenation of alkenes in water. The absence of metallic catalyst and the release of N_2_, and partly H_2_ (which can be possibly both captured and recombined to NH_3_), as sole co‐products represent noticeable advantages of this technology. Furthermore, the low temperature of the ultrasonic reactor (30 °C) is of high interest in terms of selectivity as no other carbon‐based product than alkanes were formed (no formation of alkyl amines or alkyl alcohols).

In terms of perspective, our experiments strongly suggest that switching from a batch to a continuous flow reactor and the coupling of ultrasound with current catalytic technologies utilizing N_2_H_4_ as a reductant offer opportunities to optimize the energy expenditure of this technology. The Agence Nationale de la Recherche (project JCJC ANR‐20‐CE07‐0006) is acknowledged for financial support

## Conflict of interest

The authors declare no conflict of interest.

## Supporting information

As a service to our authors and readers, this journal provides supporting information supplied by the authors. Such materials are peer reviewed and may be re‐organized for online delivery, but are not copy‐edited or typeset. Technical support issues arising from supporting information (other than missing files) should be addressed to the authors.

Supporting Information

Supporting Information

Supporting Information

Supporting Information

Supporting Information

Supporting Information

## Data Availability

The data that support the findings of this study are available from the corresponding author upon reasonable request.
