# Supplementary material for: Sonochemically‐Induced Reduction of Alkenes to Alkanes with Ammonia
Source: Angew Chem Int Ed Engl. 2022 Nov 15;61(51):e202212719. doi: 10.1002/anie.202212719 (PMC10099763; doi:10.1002/anie.202212719)
Supplement: Supplementary file 1 — Supporting Information [file ANIE-61-0-s002.pdf]

Angewandte Chemie
Eine Zeitschrift der Gesellschaft Deutscher Chemiker

Supporting Information

Sonochemically-Induced Reduction of Alkenes to Alkanes with Ammonia

*A. Humblot, T. Chave, P. N. Amaniampong, S. Streiff, F. Jérôme**

Materials and Methods

Experimental procedure for the sonochemically-induced reduction of alkenes to alkanes with ammonia

To a solution of alkene in dichloromethane (5 mL at 10^{-2} M) was added the corresponding amount of activated carbon (DARCO®-100) to get the desired loading. Characterization of the activated carbon are provided below in Table S1. The mixture was stirred for 10 min and dichloromethane was then evaporated under vacuum affording a black powder of activated carbon impregnated with the alkene. In most of reactions presented in the manuscript, 5 wt% of 1-octene were loaded on activated carbon. Then, 100 mg of activated carbon impregnated with the alkene and 100 mL of an aqueous solution of NH_3 (5 wt%) were introduced into the ultrasonic reactor. The suspension was then mechanically stirred and sonicated at 525 kHz for the desired time. The temperature of the reactor was continuously maintained at 30 °C with a cooling liquid system. During ultrasonic irradiation, air was also continuously blown in the cup-horn to cool down the piezoelectric ceramics (Fig. S2).

At the end of the reaction, the solution was filtered off to recover the activated carbon. The carbon was then washed with 2 mL of dichloromethane to desorb organic products and the dichloromethane phase was analyzed by Gas Chromatography with Flame Ionization Detection (GC-FID).

Note 1: One should note that a carbonaceous support has been preferred over inorganic supports such as silica gel or alumina, these latter being partly dissolved in aqueous NH_3 solution under ultrasonic irradiation. In addition, with hydrophilic surfaces (silica or alumina), we observed a solvation with water leading to an uncontrolled desorption of 1-octene from the inorganic support during the reaction.

Note 2: ultrasonic reactions with 1-octene loaded on carbon were repeated 10 times to check the repeatability. We observed a yield variation of ±5%

Calibration of 1-octene and n-octane in GC-FID

Standard solutions of 1-octene and *n*-octane with concentration ranging from 0.1 to 2 g/L (from 1 to 19 mmol/L) in dichloromethane were prepared, using *n*-dodecane as an internal standard at a concentration of 44 mmol/L (*i.e*. before analysis 10 µL of *n*-dodecane was added in 1 mL of aliquots). The samples were analyzed on a Bruker GC-FID equipped with a HP-5MS column. The injection volume was 1 µL and the flow rate 1 mL/min. The injector temperature was set at 270 °C and the detector at 300 °C. The heating ramp of the oven is provided in Fig. S3. The calibration curves of 1-octene and *n*-octane are provided in Fig. S4 and S5. A similar procedure was employed for other alkenes presented in the Table 2 of the main manuscript.

Supplementary Text

Experiment in a polyethylene flask

In order to rule out a possible role of the stainless steel reactor wall on the reaction mechanism, the reaction was conducted in a polyethylene flask immerged into the ultrasonic reactor (Fig. S11). A polyethylene flask was selected as it is transparent to ultrasonic waves. Typically, the ultrasonic reactor was filled with 75 mL of water and the polyethylene flask was filled with 25 mg of 5 wt% 1-octene supported on activated carbon and 25 mL of aqueous NH_3 (5 wt%) solution. The polyethylene flask was then immerged into the ultrasonic reactor. As above mentioned, the temperature of the ultrasonic reactor was maintained at 30 °C with a cooling liquid system and the solution was continuously sonicated at 525 kHz. At the end of the reaction, the solution was filtered off to recover the activated carbon. The carbon was then washed with dichloromethane to desorb organic products from the surface of the carbon, and the dichloromethane phase was analyzed by GC-FID. Under these reaction conditions, the yield to *n*-octane was 36% (*vs* 33% without the polyethylene flask). These results suggest that the stainless steel reactor wall do not participate, at least significantly, to the reaction mechanism.

Note 1: In contrast to the previous protocol, it was not possible to mechanically stir the solution into the polyethylene flask, but this had no real impact on the efficiency of the reaction. Indeed, when the reaction was directly conducted in the ultrasonic reactor, we observed that similar results were obtained with or without stirring, which can be explained by the ability of ultrasonic waves to stir the solution.

Note 2: The presence of metallic species in the DARCO®-100 activated carbon has been also checked by X-ray fluorescence. Presence of metals has been detected in trace amount (Fe: 3 ppm; Ti: 1.6 ppm;

Cr, Cu, Zn, Sr and Pd: < 0.1 ppm). Even if these metallic species were detected as trace, it is impossible to completely rule out their participation in the reaction mechanism. That is the reason why we did not claim "metal-free reaction" in the main article.

Illustration of a possible coupling of ultrasound with catalysis

Ultrasonication at 525 kHz of a suspension of 100 mg of activated carbon coated with 5 wt% of 1-octene in 100 mL of aqueous NH_3 (5 wt%) and under bubbling of Ar (80 mL/min of argon) led to 33% conversion of 1-octene to *n*-octane after 5 hours. At these 5 h of ultrasonic irradiation, the amount of free hydrazine accumulated into the reactor correspond to 1.3 eq of remaining 1-octene. Using flavin as a reference catalyst, it should be theoretically possible to terminate the hydrogenation of 1-octene under silent conditions as reported in ref 23-26 in the main manuscript (Fig. S17).

If the amount of carbon coated with 5 wt% of 1-octene is decreased from 100 to 50 mg, the ultrasonic irradiation could be stopped after only 2 h (21% conv) before completion of the hydrogenation with flavin and *in situ* released free N_2H_4.

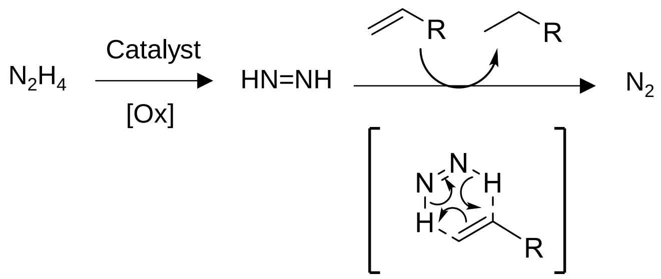

Fig. S1. Accepted mechanism for the N_2H_4-mediated reduction of alkenes.

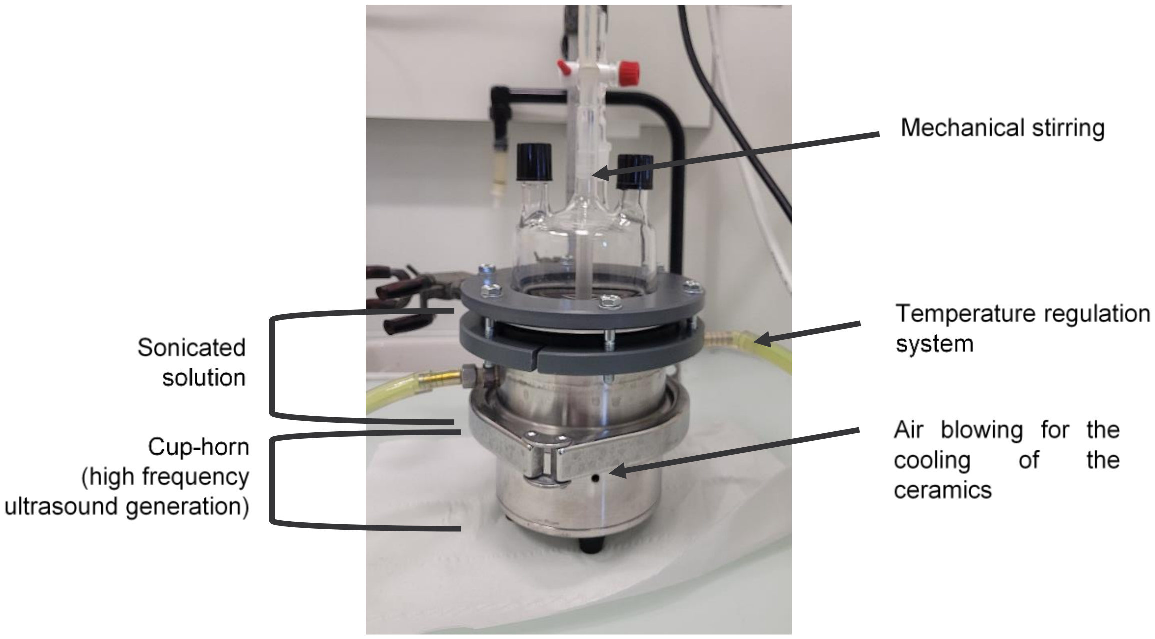

Fig. S2. Picture of the high frequency ultrasonic reactor (from Sinaptec) used in this study**.**

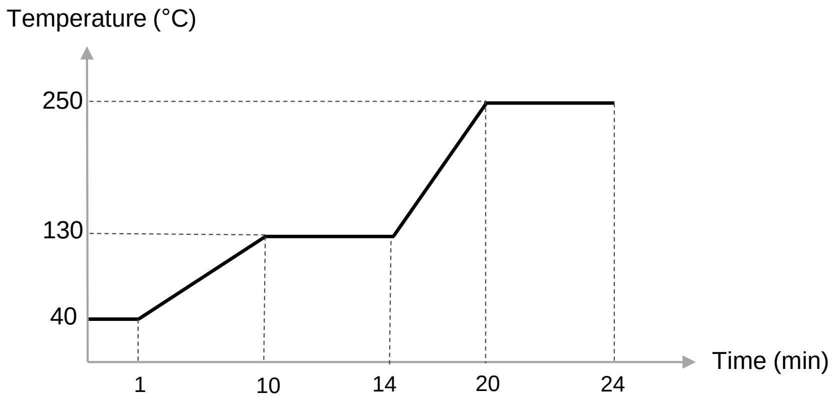

Fig. S3. GC oven heating ramp

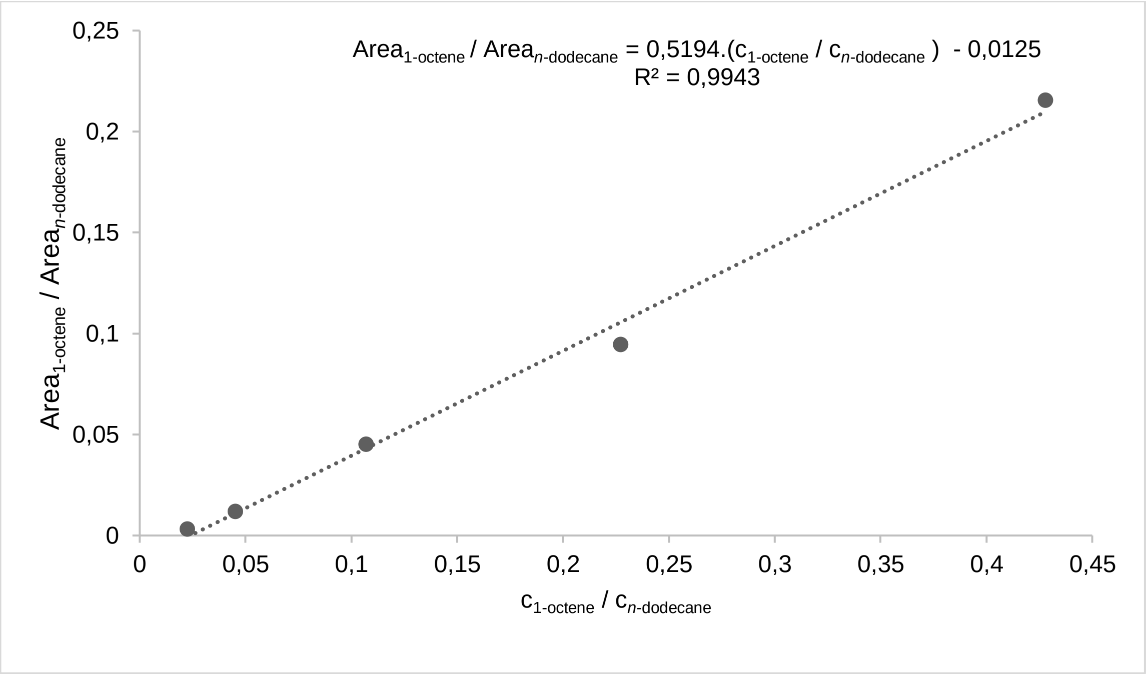

Fig. S4. Calibration curve of 1-octene in GC-FID, using *n*-dodecane as an internal standard

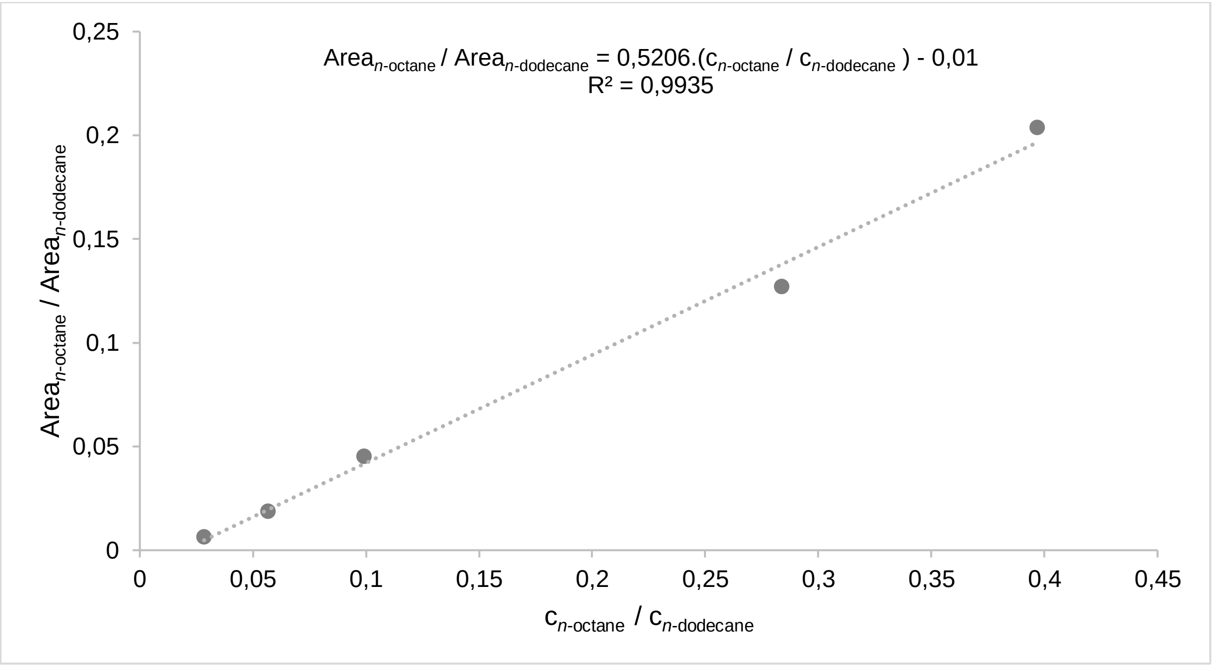

Fig. S5. Calibration curve of *n*-octane in GC-FID, using *n*-dodecane as an internal standard

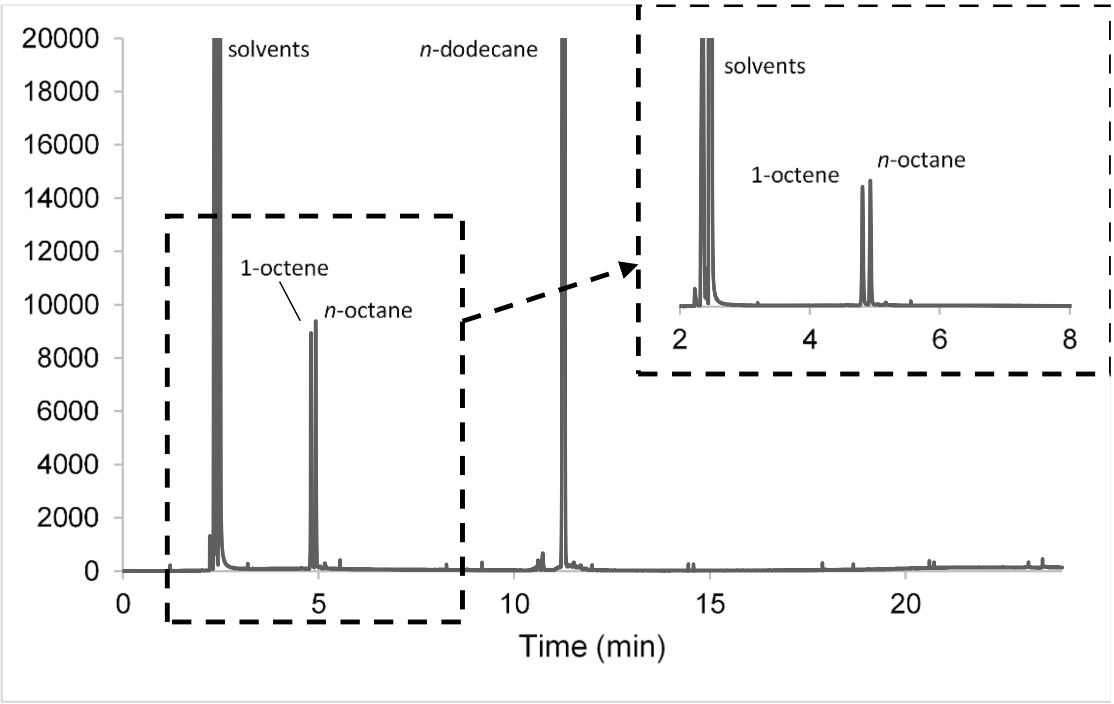

Fig. S6. Example of GC chromatogram obtained after washing the activated carbon loaded with 5 wt% of 1-octene with dichloromethane. (5 wt% of 1-octene supported on 50 mg of activated carbon, 100 mL 5 wt% ammonia solution, 525 kHz, 30 °C, 6 h, under air).

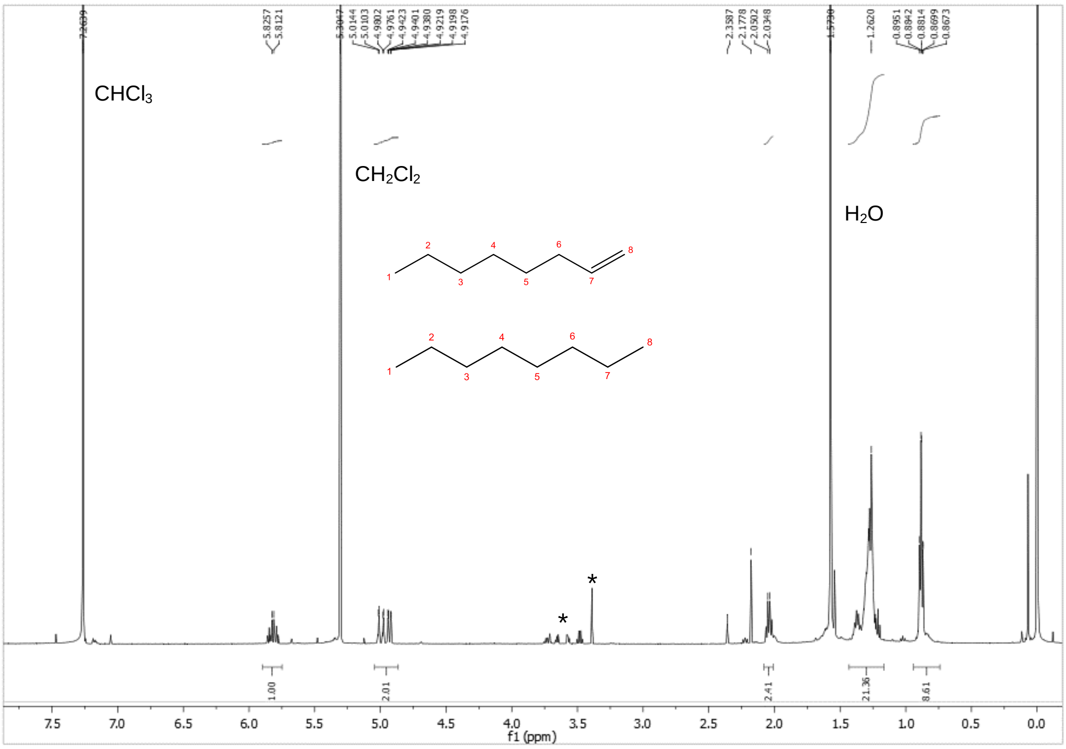

* *Pollution of the NMR tube with diglyme*

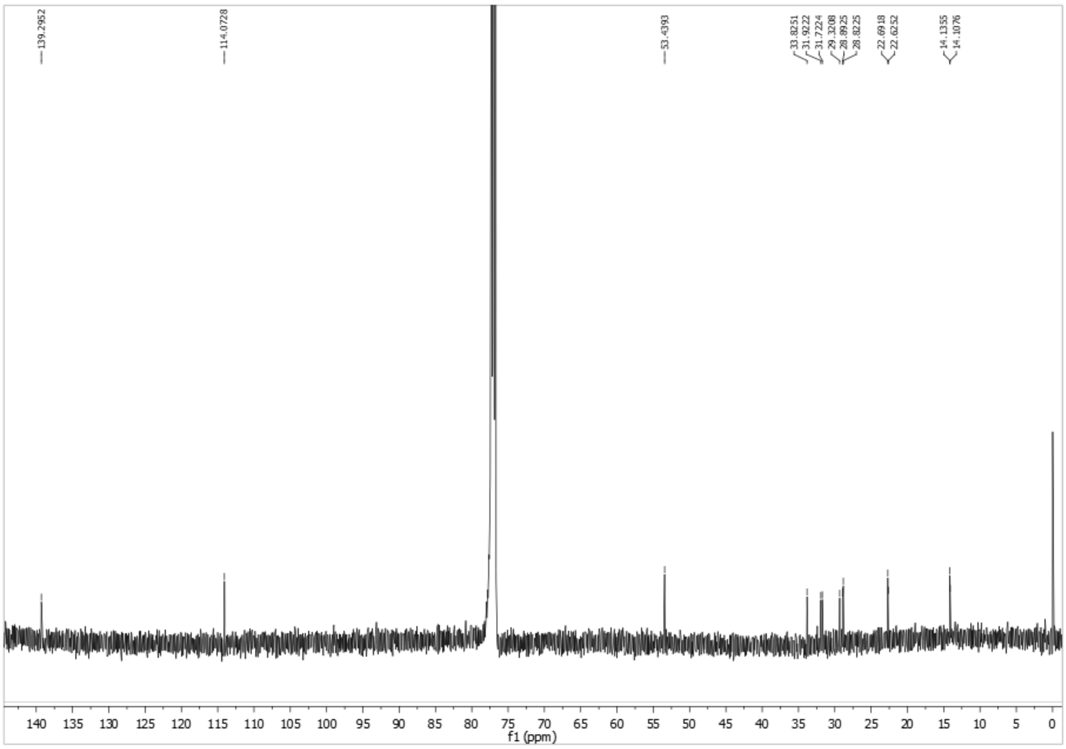

Fig. S7. ^{1}H and ^{13}C NMR (500 MHz, CDCl3) of the products of the reaction after washing the activated carbon with dichloromethane. (5 wt% of 1-octene supported on 50 mg of activated carbon, 100 mL 5 wt% ammonia solution, 525 kHz, 30 °C, 6 h, under air)

1-octene: ^{1}H NMR (500 MHz, $CDCl_3$) δ 5.81 ppm (m, $1H_7$), 4.97 ppm (dm, $1H_{8trans}$, ^{3}J = 17 Hz), 4.91 ppm (dm, $1H_{8cis}$, ^{3}J = 10 Hz) 2.01 ppm (m, $2H_6$), 1.26 ppm (m, $8H_{2-5}$), 0.88 ppm (t, $3H_1$, ^{3}J = 7 Hz).
$^{13}C\{^{1}H\}$ NMR (125 MHz, $CDCl_3$) δ 139.3 (C_7), 114.1 (C_8), 33.8 (C_6), 31.7 (C_3), 28.9 (C_5), 28.8 (C_4), 22.6 (C_2), 14.1 (C_1)
HRMS (GC-QTOF) m/z: Calcd for C_8H_{16} 112.1252. Found 112.1249.

n-octane: ^{1}H NMR (500 MHz, $CDCl_3$) δ 1.26 ppm (m, $12H_{2-7}$), 0.88 ppm (t, $6H_{1,8}$, ^{3}J = 7 Hz).
$^{13}C\{^{1}H\}$ NMR (125 MHz, $CDCl_3$) δ 31.9 ($C_{3,6}$), 29.3 ($C_{4,5}$), 22.7 ($C_{2,7}$), 14.1 ($C_{1,8}$)
HRMS (GC-QTOF) m/z: Calcd for C_8H_{18} 114.1409. Found 114.1400

The *n*-octane yield is 51% by GC-FID and 48% by ^{1}H NMR.

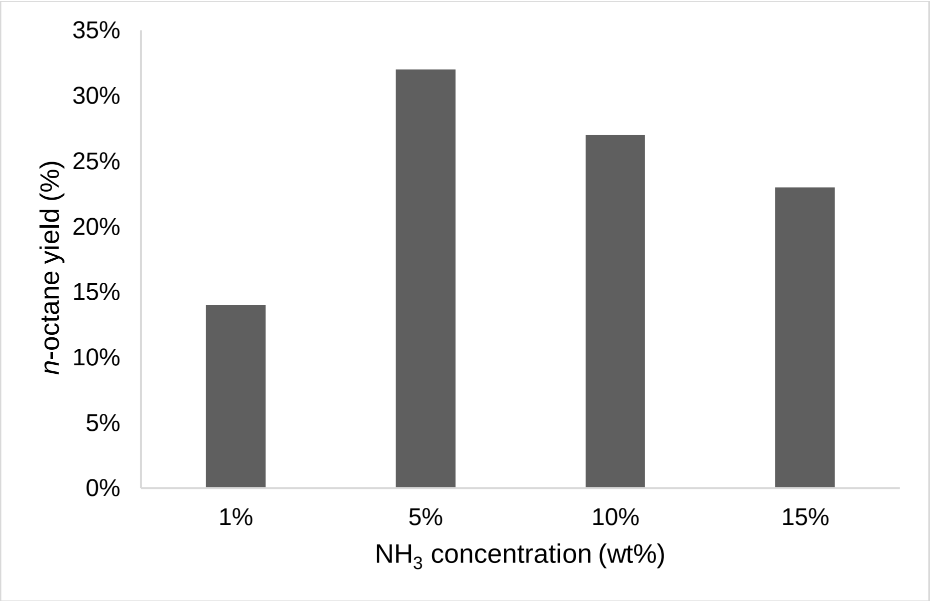

Fig. S8. Effect of the aqueous NH_3 concentration on the *n*-octane yield (5 wt% of 1-octene supported on 100 mg of activated carbon, 100 mL of aqueous NH_3 solution, 525 kHz, 30 °C, 6 h, under air)

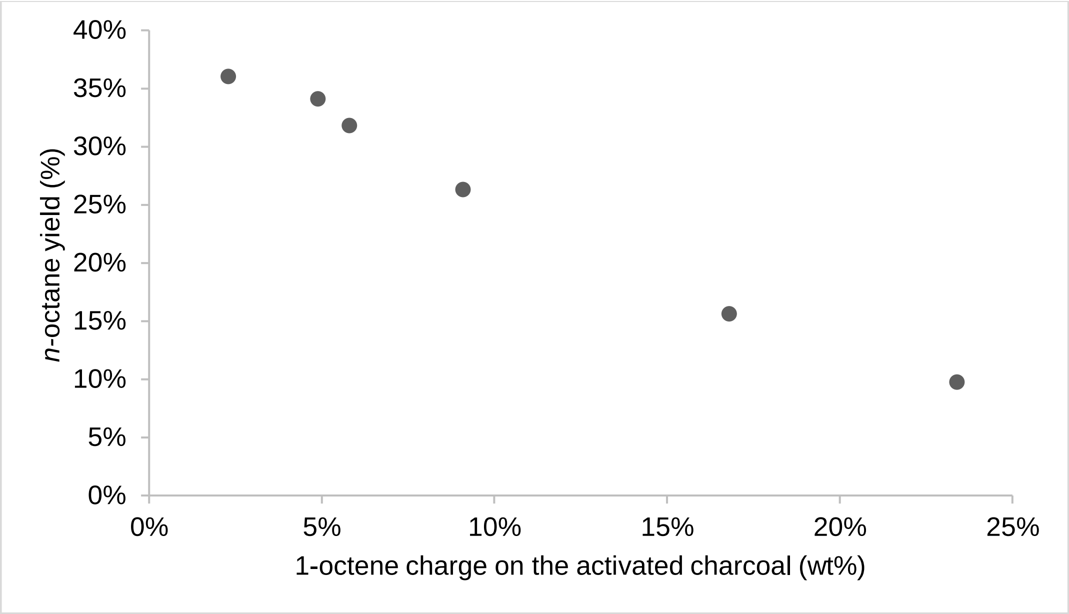

Fig. S9. Effect of the quantity of 1-octene loaded on activated carbon on the *n*-octane yield (2-23 wt% of 1-octene supported on 100 mg of activated carbon, 100 mL of aqueous NH_3 (5 wt%), 525 kHz, 30 °C, 6 h, under air)

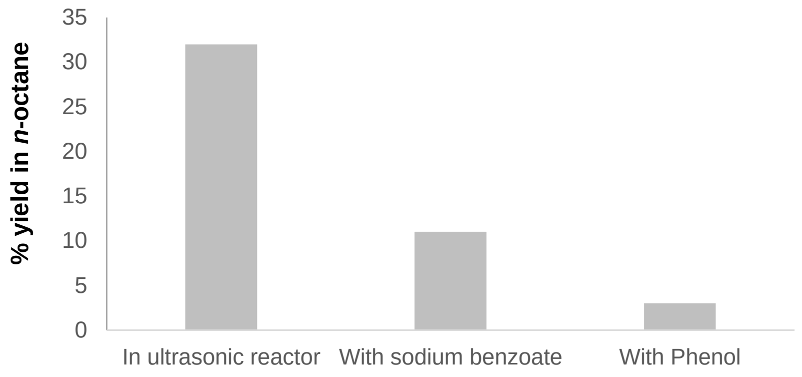

Fig. S10. Effect of radical scavenger (0.1 M sodium benzoate and phenol) on the *n*-octane yield (6 h, 525 KHz, 30 °C, under air).

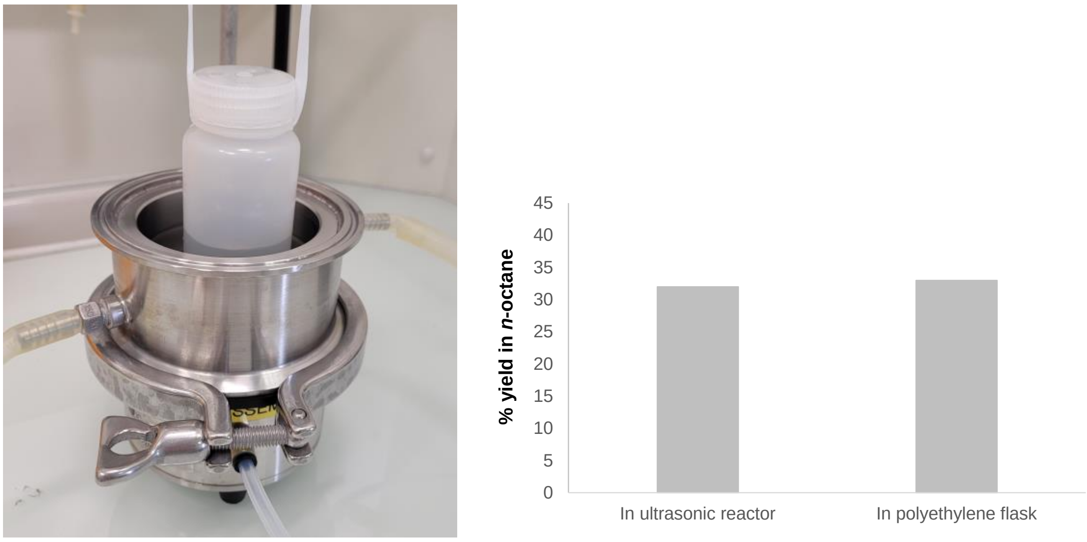

Fig. S11. Compartmentation of the ultrasonic irradiation in a polyethylene flask (525 kHz, 6 h, in 5 wt% aqueous NH_3, 30 °C, under air).

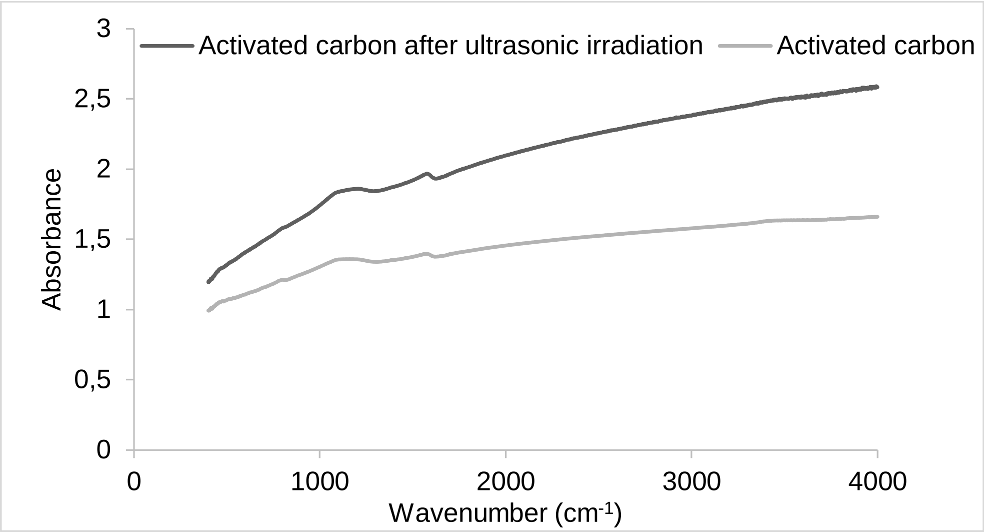

Fig. S12. FTIR spectra of fresh activated carbon and activated carbon after ultrasonic irradiation at 525 kHz in 5 wt% aqueous NH_3 for 6 h.

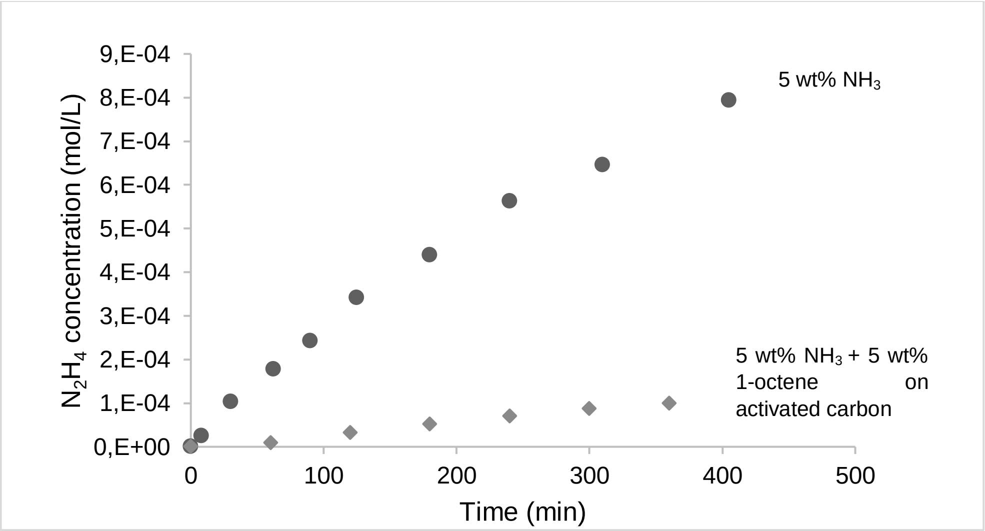

Fig. S13. Plot of the hydrazine concentration as a function of the ultrasonic time (in 5 wt% NH_3 with and without activated carbon coated with 5 wt% 1-octene, under air, 30 °C, 525 kHz).

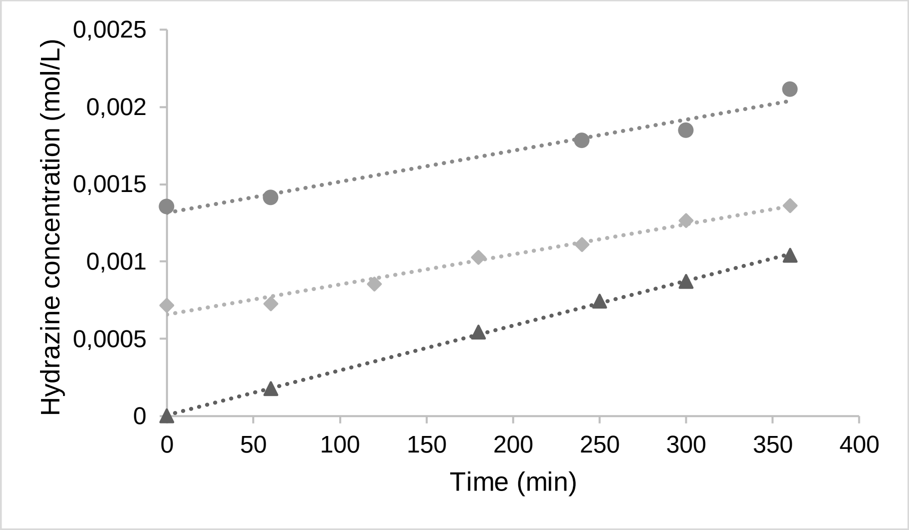

Fig. S14. Hydrazine production rate at different initial amount of hydrazine (in 5 wt% NH_3 with 50 mg 5 wt% 1-octene on activated carbon, 525 KHz, under air, 30 °C).

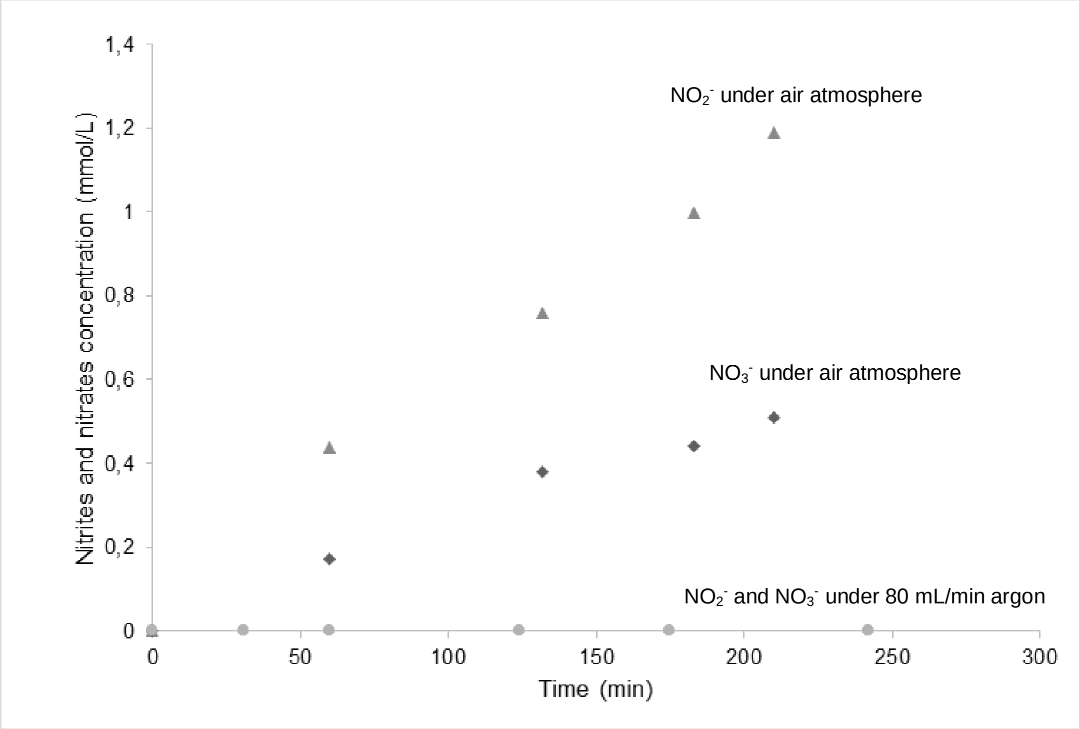

Fig. S15. Effect of the atmosphere (air, argon) on the nitrites (NO_2^-) and nitrates (NO_3^-) formation (5 wt% of alkene supported on 50 mg of activated carbon, 100 mL of aqueous NH_3 solution (5 wt%), 525 kHz, 30 °C, 6 h, under air or argon at 80 mL/min). The amount of nitrites/nitrates was determined using ion chromatography (IonPac column, 3x150 mm, eluent: 8.0 mM Na_2CO_3/1.0 mM $NaHCO_3$).

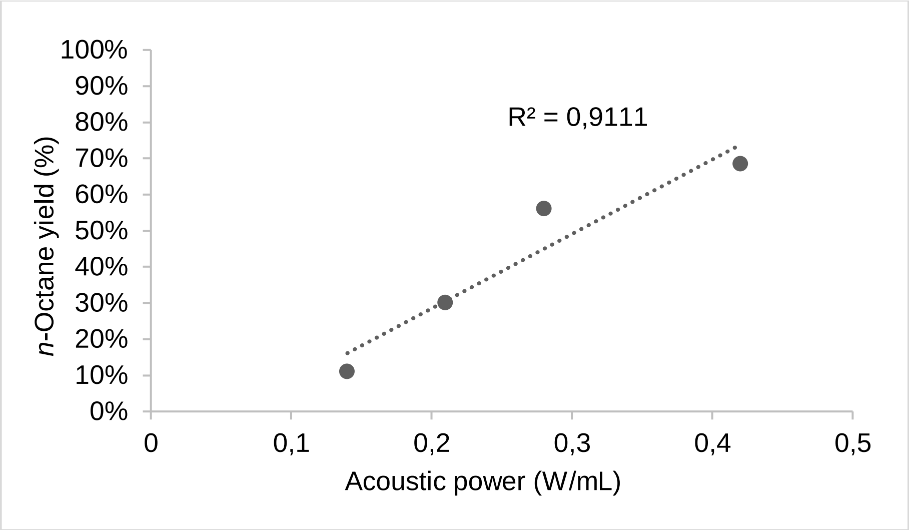

Fig. S16. Effect of the acoustic power (volume of solution) on the *n*-octane yield (5 wt% of 1-octene supported on activated carbon, 5 wt% ammonia solution, 525 kHz, 30 °C, 6 h, under air).

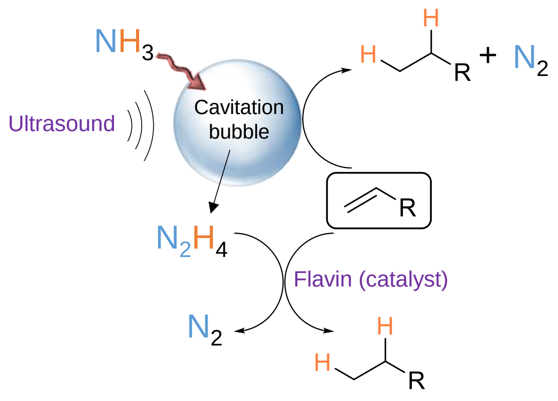

Fig. S17. Schematic representation of a coupling of ultrasound with current catalytic routes based on the use of flavins

Table S1. Characterization of carbon materials used in this study

Activated carbon	BET (m^2/g)	% micropore	Average pore width (4V/A by BET) (nm)	micropore volume (cm^3/g)	mesopore volume (cm^3/g)	Yield[a] (%)
**Darco®-100**	511	87.3	1.82	0.251	-	32
**Nuchar® SA-20**	1651	5	3.86	-	1.07	20

[a] 525 kHz, 6 h, 100 mg of activated carbon impregnated with 5 wt% of 1-octene, air, 30 °C

Table S2. *n*-octane yield after recycling of the activated carbon (5 wt% of 1-octene supported on activated carbon, 100 mL of aqueous NH_3 (5 wt%), 525 kHz, 30 °C, 6 h, under air).

Activated carbon	*n*-octane yield (%) ±5%
Fresh activated carbon	33%
After 3 uses	28%

Between each run, the activated carbon was washed with dichloromethane to desorb all organic products and then water and ethanol. It was then dried in the oven at 50 °C for 15 h before being impregnated again with 1-octene.

Additional figures-$^{1}H/^{13}C$ NMR and GC analyses

n-alkanes described in the manuscript are bulk chemicals widely processed on a large scale. Full characterization of these chemicals is widely available in the literature. Below, we show the GC and NMR profiles of the crude reaction media in order to show the high selectivity of this sonochemically-induced reduction of alkene with NH_3. Yields were calculated either by GC or ^{1}H NMR (based on integration of the $-CH_3$ group)

1-heptene reduction

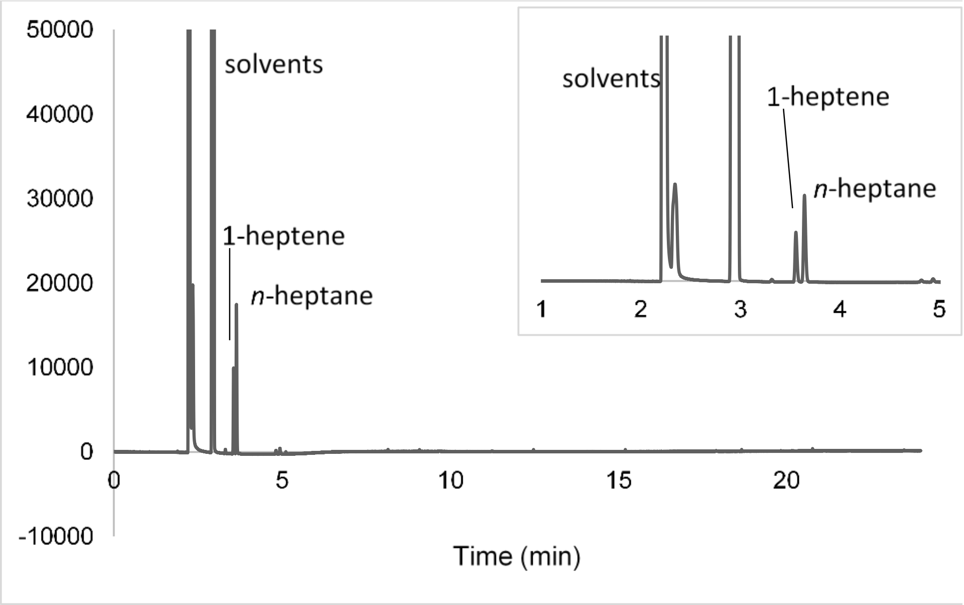

Fig. S18. GC chromatogram of 1-heptene and *n*-heptane after the reaction (5 wt% of 1-heptene supported on 50 mg of activated carbon, 100 mL 5 wt% ammonia solution, 525 kHz, 30 °C, 6 h, under air).

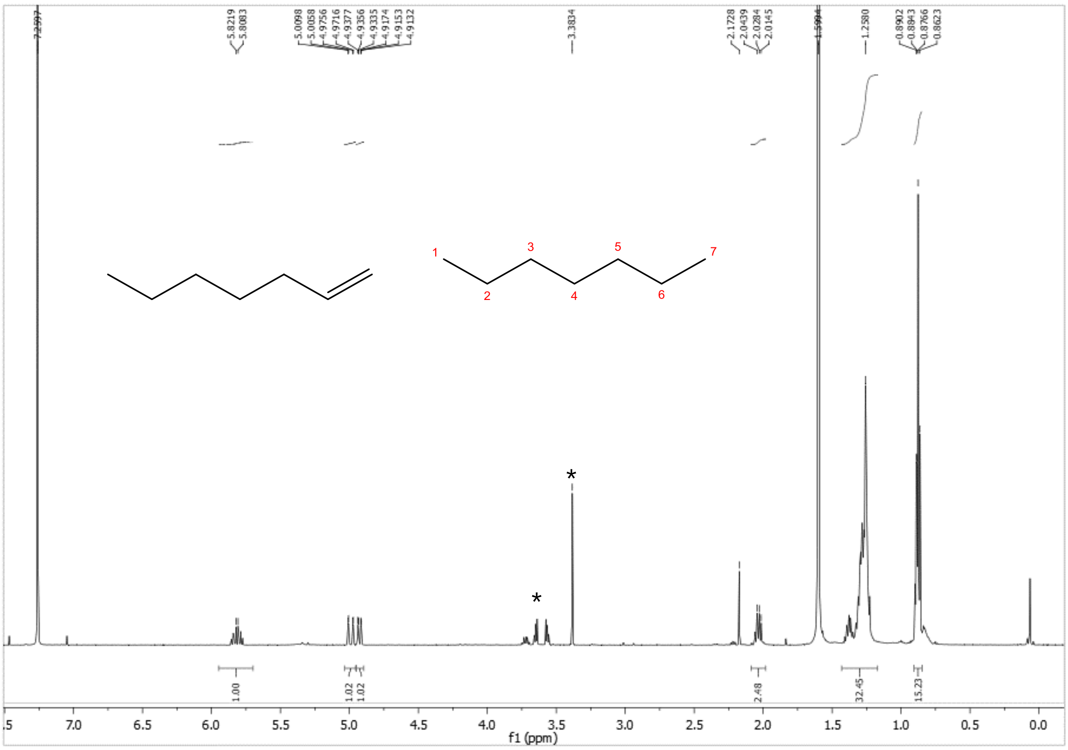

* Pollution of the NMR tube with diglyme

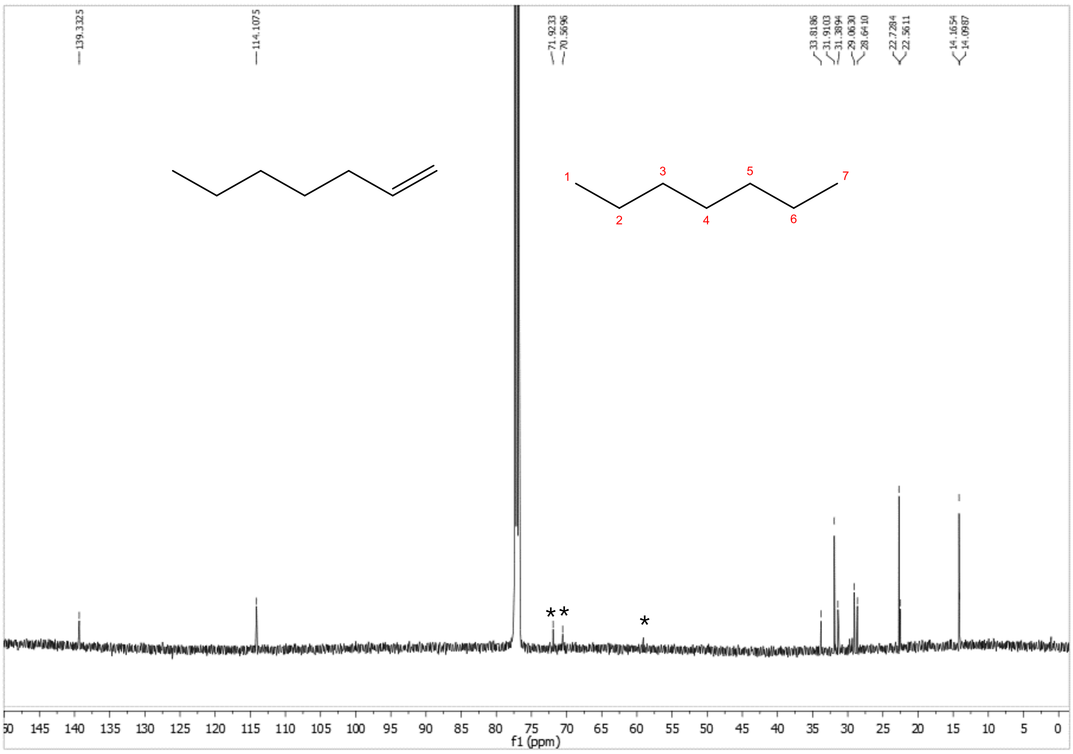

* *Pollution of the NMR tube with diglyme*

Fig. S19. ^{1}H and ^{13}C NMR (500 MHz, $CDCl_3$) of 1-heptene and *n*-heptane after the reaction. (5 wt% of 1-heptene supported on 50 mg of activated carbon, 100 mL 5 wt% ammonia solution, 525 kHz, 30 °C, 6 h, under air).

*n*-heptane: ^{1}H NMR (500 MHz, $CDCl_3$) δ 1.26 ppm (m, $10H_{2\text{-}6}$), 0.88 ppm (t, $6H_{1,7}$, ^{3}J = 7 Hz).$^{13}C\{^{1}H\}$ NMR (125 MHz, $CDCl_3$) δ 31.9 ($C_{3,5}$), 29.1 (C_4), 22.7 ($C_{2,6}$), 14.1 ($C_{1,7}$). HRMS (GC-QTOF) m/z: Calcd for C_7H_{16} 100.1252. Found 100.1254

Yield determined by GC and ^{1}H NMR were 65% and 67%, respectively

1-dodecene reduction

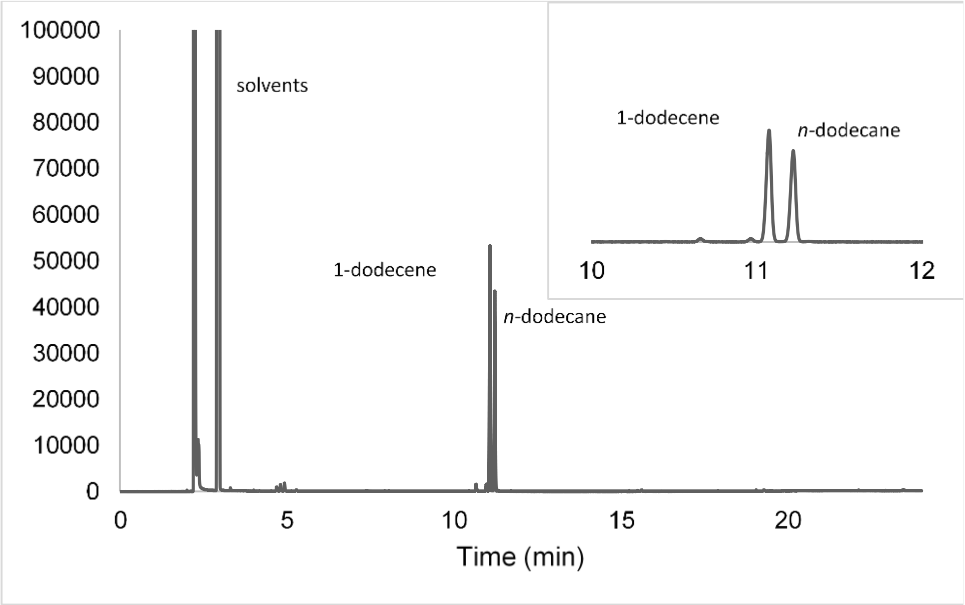

Fig. S20. GC chromatogram of 1-dodecene and *n*-dodecane after the reaction. (5 wt% of 1-dodecene supported on 50 mg of activated carbon, 100 mL 5 wt% ammonia solution, 525 kHz, 30 °C, 6 h, under air).

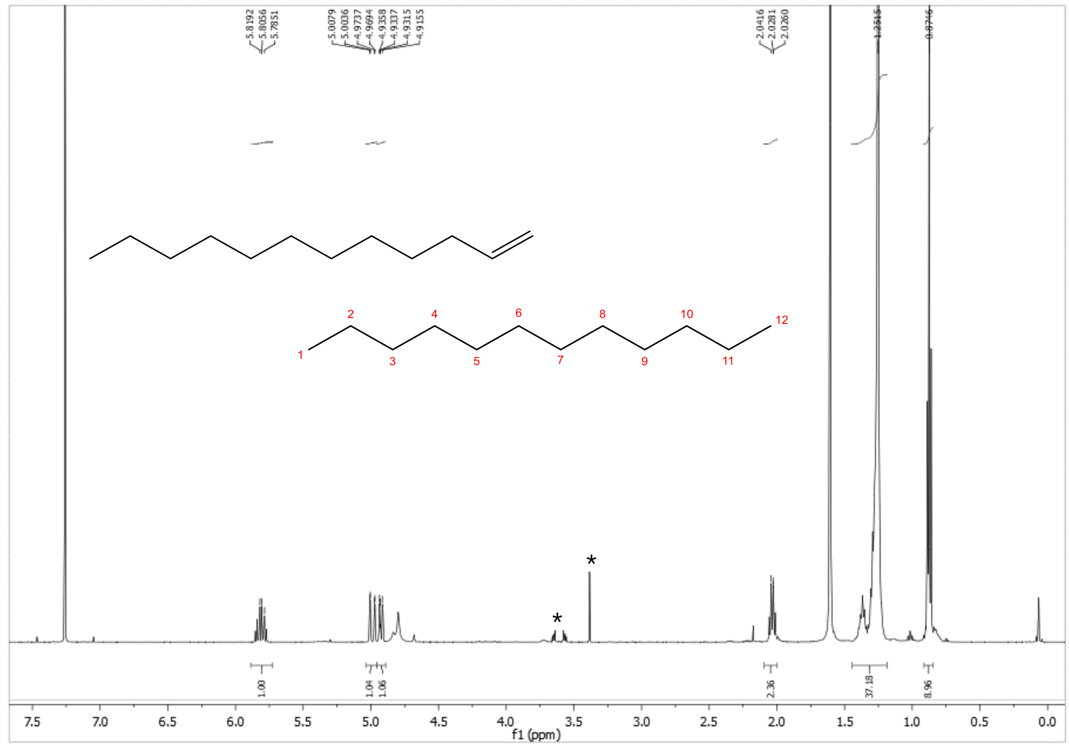

** Pollution of the NMR tube with diglyme*

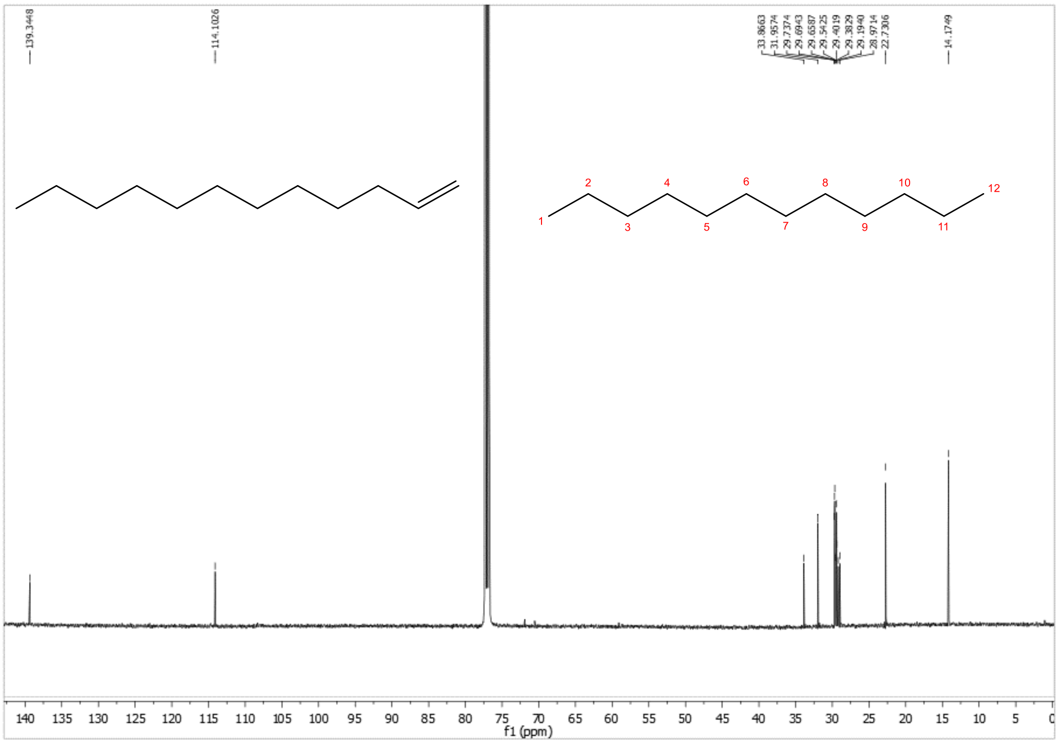

Fig. S21. ^{1}H and ^{13}C NMR (500 MHz, $CDCl_3$) of 1-dodecene and *n*-dodecane after the reaction. (5 wt% of 1-dodecene supported on 50 mg of activated carbon, 100 mL 5 wt% ammonia solution, 525 kHz, 30 °C, 6 h, under air)

<u>*n*-dodecane</u>: ^{1}H NMR (500 MHz, $CDCl_3$) δ 1.25 ppm (m, $20H_{2-11}$), 0.87 ppm (t, $6H_{1,12}$, ^{3}J = 7 Hz). $^{13}C\{^{1}H\}$ NMR (125 MHz, $CDCl_3$) δ 31.9 ($C_{3,10}$), 28.9-29.7 (C_{4-9}), 22.7 ($C_{2,11}$), 14.2 ($C_{1,12}$). HRMS (GC-QTOF) m/z: Calcd for $C_{12}H_{26}$ 170.2035. Found 170.2014.

Yield determined by GC and ^{1}H NMR were 45% and 50%, respectively.

Cyclooctene reduction

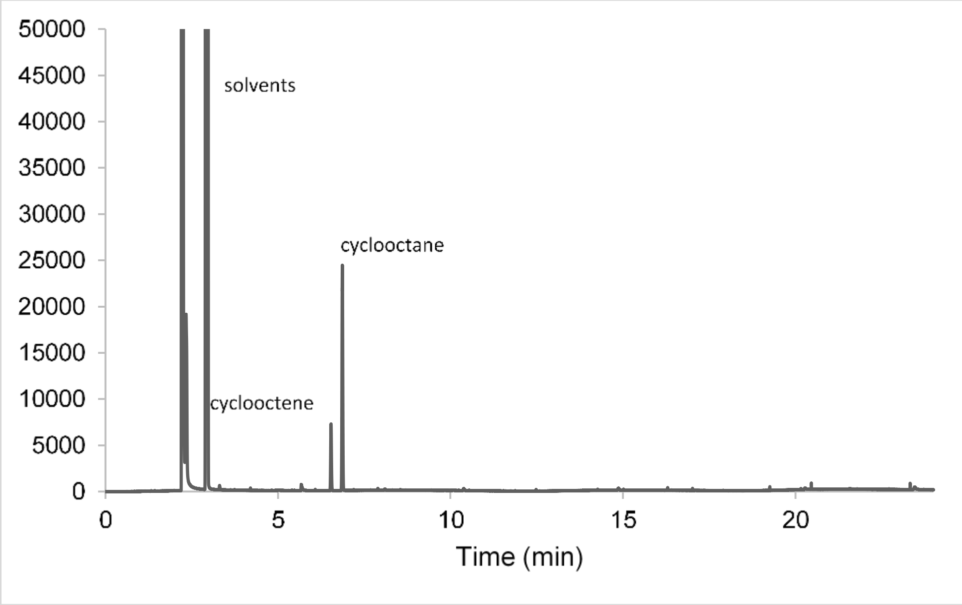

Fig. S22. GC chromatogram of cyclooctene and cyclooctane after the reaction (5 wt% of cyclooctene supported on 50 mg of activated carbon, 100 mL 5 wt% ammonia solution, 525 kHz, 30 °C, 6 h, under air).

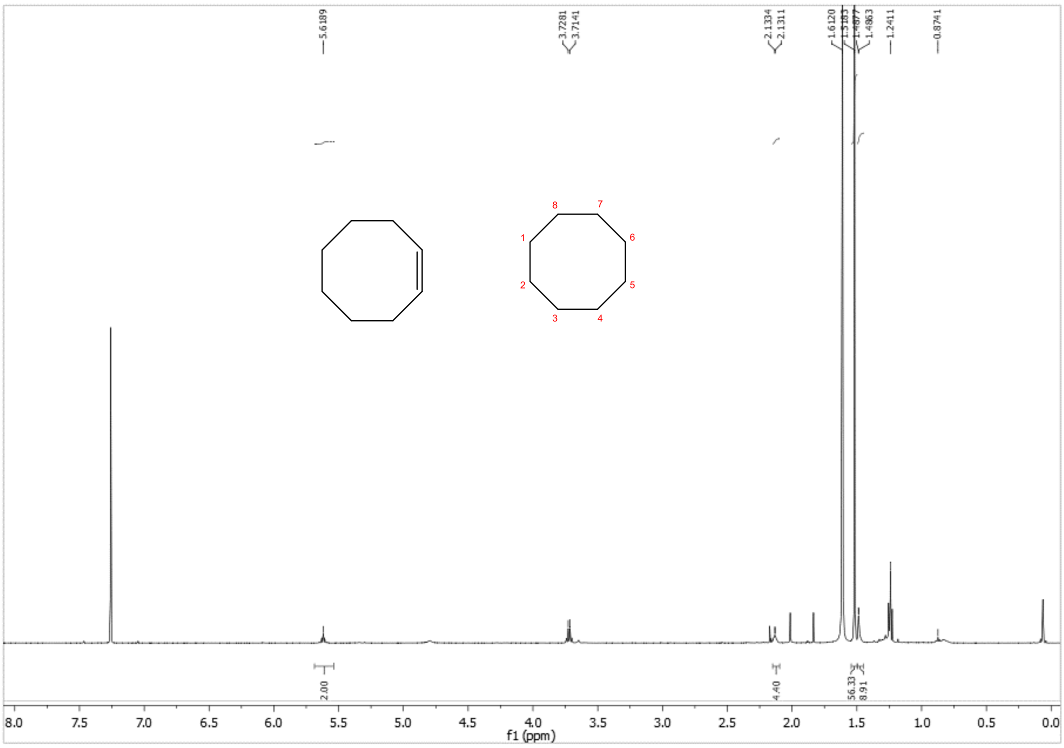

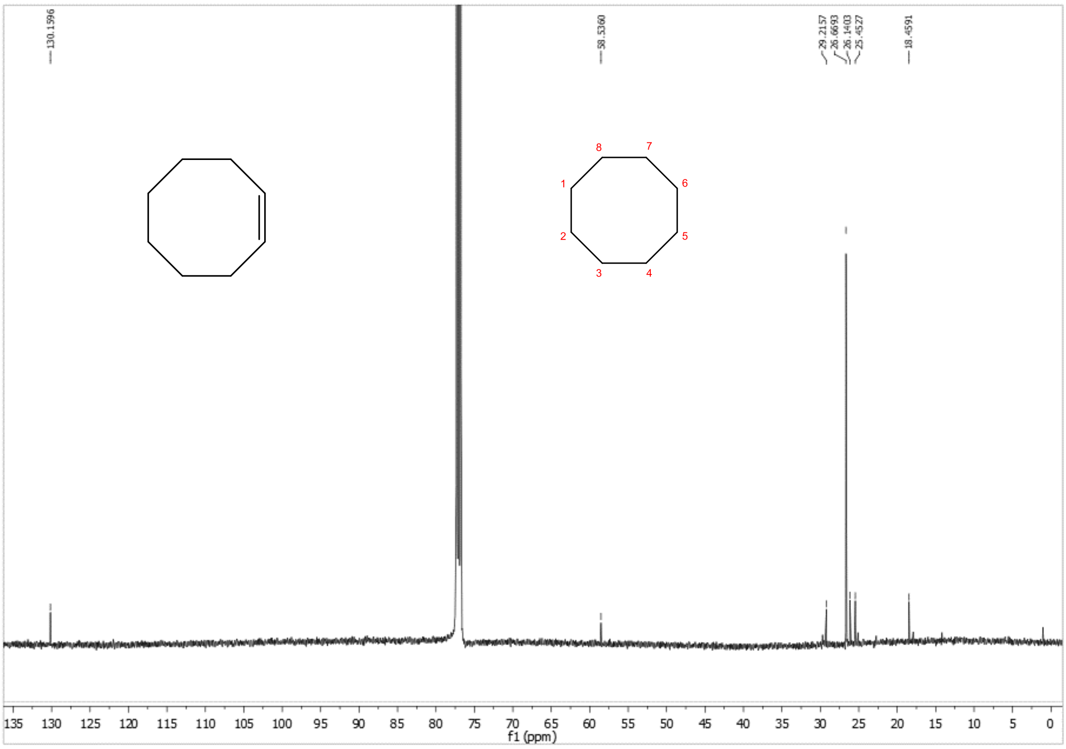

Fig. S23. ^{1}H and ^{13}C NMR (500 MHz, $CDCl_3$) of cyclooctene and cyclooctane after the reaction (5 wt% of 1-cyclooctene supported on 50 mg of activated carbon, 100 mL 5 wt% ammonia solution, 525 kHz, 30 °C, 6 h, under air)

cyclooctane: ^{1}H NMR (500 MHz, $CDCl_3$) δ 1.52 ppm (s, $16H_{1-8}$). $^{13}C\{^1H\}$ NMR (125 MHz, $CDCl_3$) δ 26.7 (C_{1-8}) HRMS (GC-QTOF) m/z: Calcd for C_8H_{16} 112.1252. Found 112.1250.

Yield determined by GC and ^{1}H NMR were 77% and 78%, respectively

Norbornene reduction

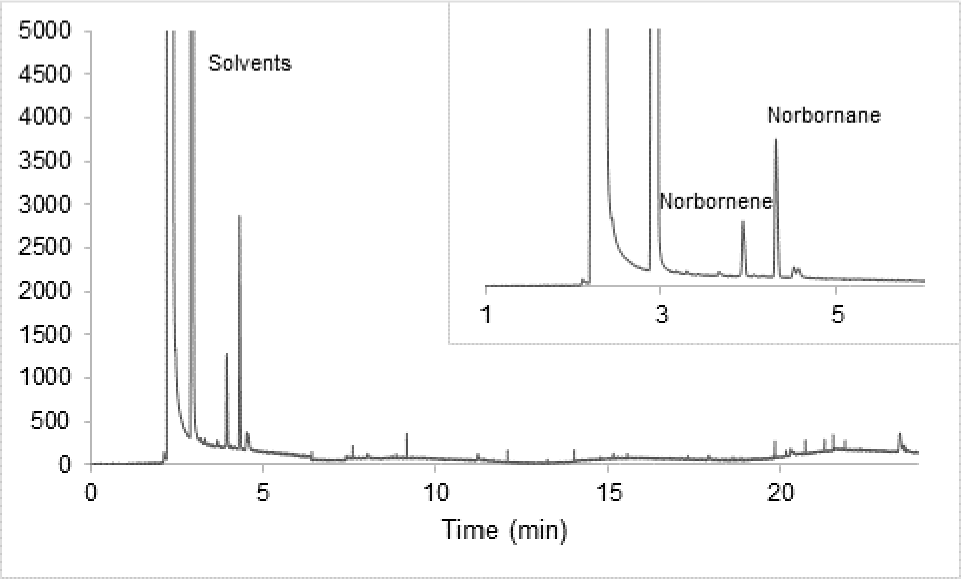

Fig. S24. GC chromatogram of norbornene and norbornane after the reaction. (5 wt% of norbornene supported on 50 mg of activated carbon, 100 mL 5 wt% ammonia solution, 525 kHz, 30 °C, 6 h, under air).

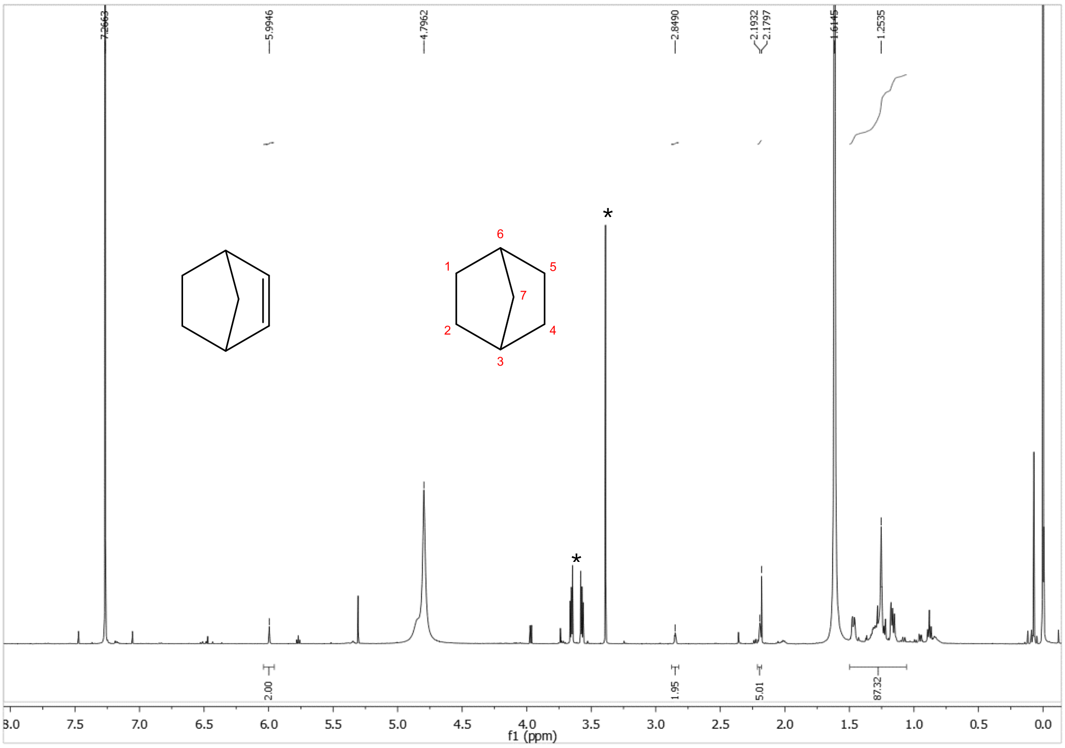

* Pollution of the NMR tube with diglyme

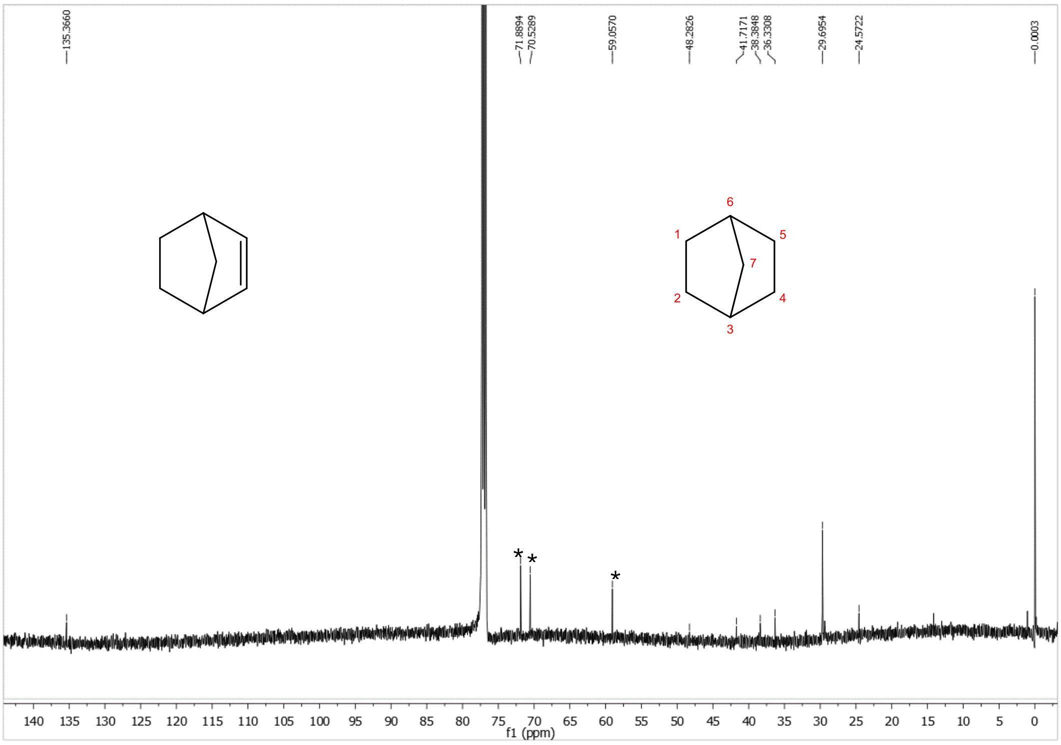

* Pollution of the NMR tube with diglyme

Fig. S25. ^{1}H and ^{13}C NMR (500 MHz, $CDCl_3$) of norbornene and norbornane after the reaction. (5 wt% of norbornene supported on 50 mg of activated carbon, 100 mL 5 wt% ammonia solution, 525 kHz, 30 °C, 6 h, under air).

norbornane: ^{1}H NMR (500 MHz, $CDCl_3$) δ 2.19 (m, $2H_{3,6}$), 1-1.50 ppm (m, $10H_{1,2,4,5,7}$). $^{13}C\{^{1}H\}$ NMR (125 MHz, $CDCl_3$) δ 38.4 (C_7), 36.3 ($C_{3,6}$), 29.7 ($C_{1,2,4,5}$). HRMS (GC-QTOF) m/z: Calcd for C_7H_{12} 96.0939. Found 96.0910.

Yield in norbornane determined by GC and ^{1}H NMR were 71% and 71%, respectively

1,7-octadiene reduction

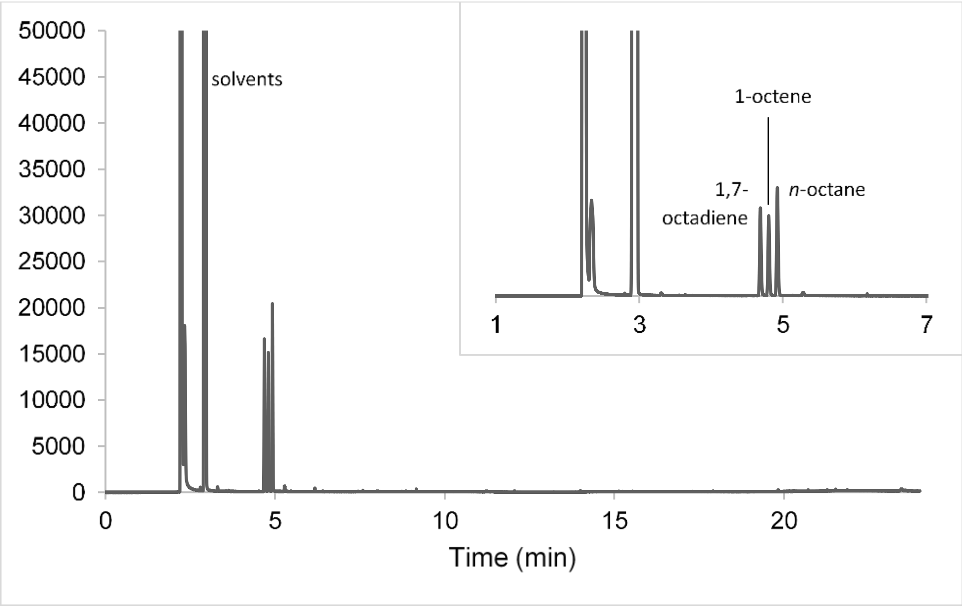

Fig. S26. GC chromatogram of 1,7-octadiene, 1-octene and *n*-octane after the reaction (5 wt% of 1,7-octadiene supported on 50 mg of activated carbon, 100 mL 5 wt% ammonia solution, 525 kHz, 30 °C, 6 h, under air).

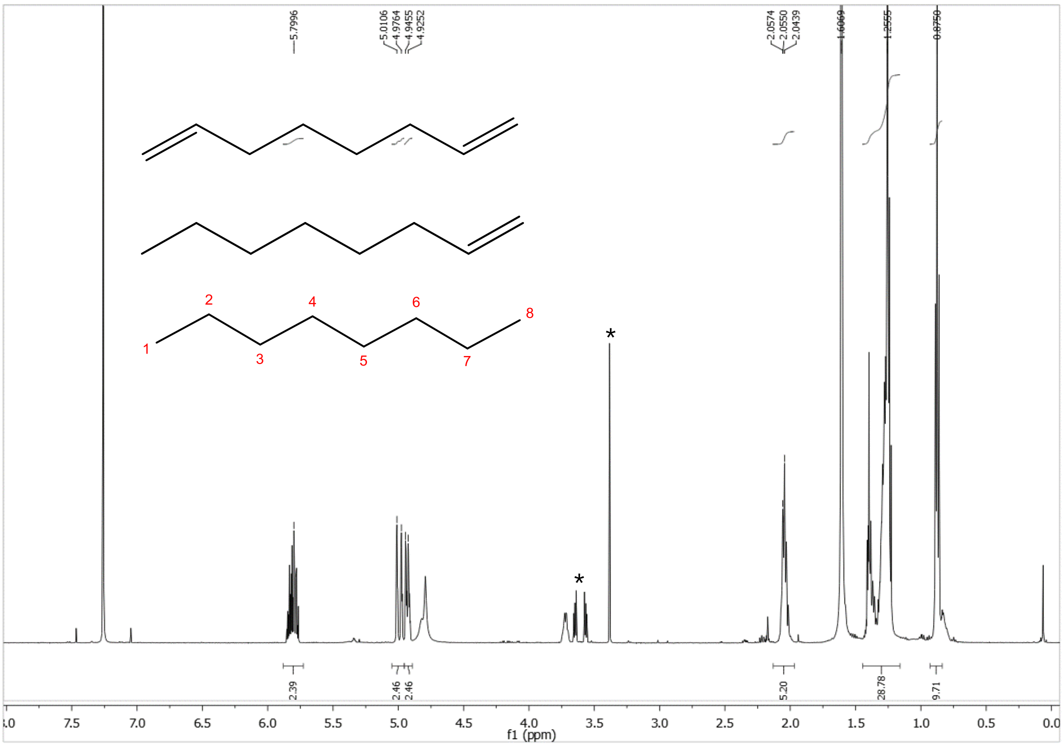

* Pollution of the NMR tube with diglyme

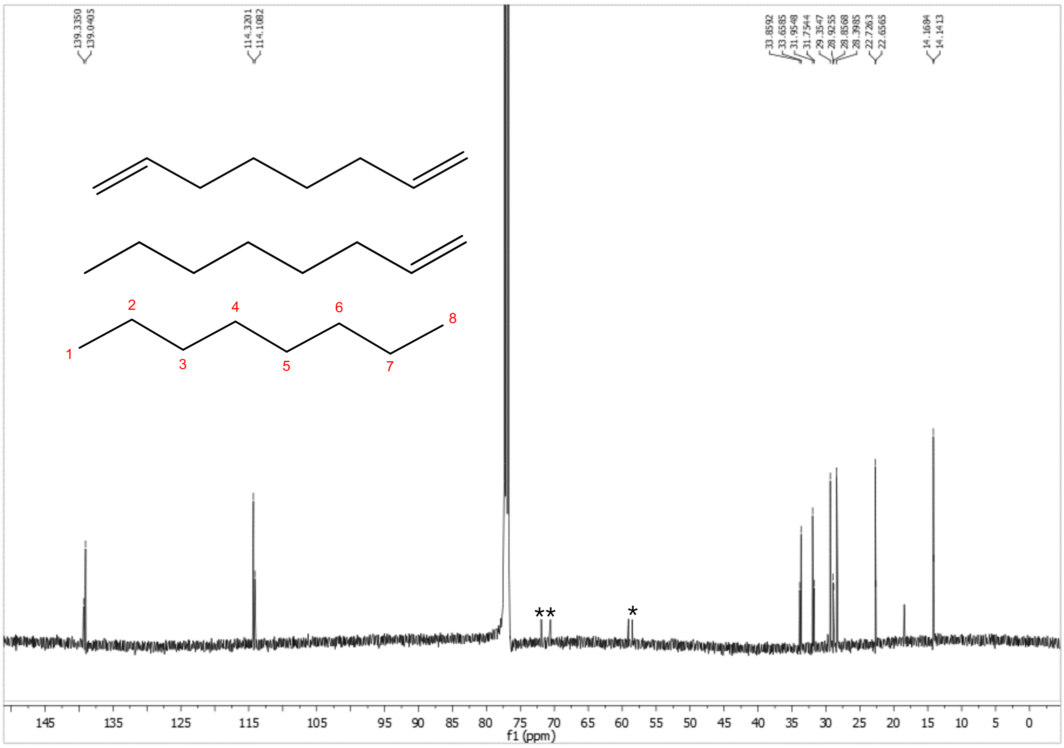

* Pollution of the NMR tube with diglyme

Fig. S27. ^{1}H and ^{13}C NMR (500 MHz, $CDCl_3$) of 1,7-octadiene, 1-octene and *n*-octane after the reaction (5 wt% of 1,7-octadiene supported on 50 mg of activated carbon, 100 mL 5 wt% ammonia solution, 525 kHz, 30 °C, 6 h, under air).

Yield in *n*-octane determined by GC and ^{1}H NMR were 40% and 43%, respectively

Yield in 1-octene determined by GC and ^{1}H NMR were 29% and 28%, respectively

2-octene reduction

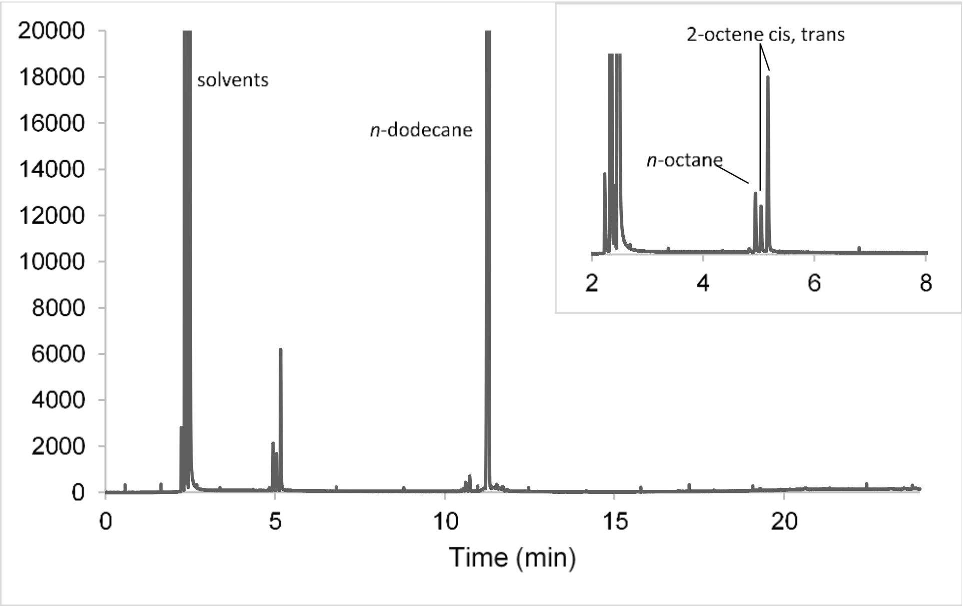

Fig. S28. GC chromatogram of 2-octene and *n*-octane after the reaction (5 wt% of 2-octene supported on 50 mg of activated carbon, 100 mL 5 wt% ammonia solution, 525 kHz, 30 °C, 6 h, under air).

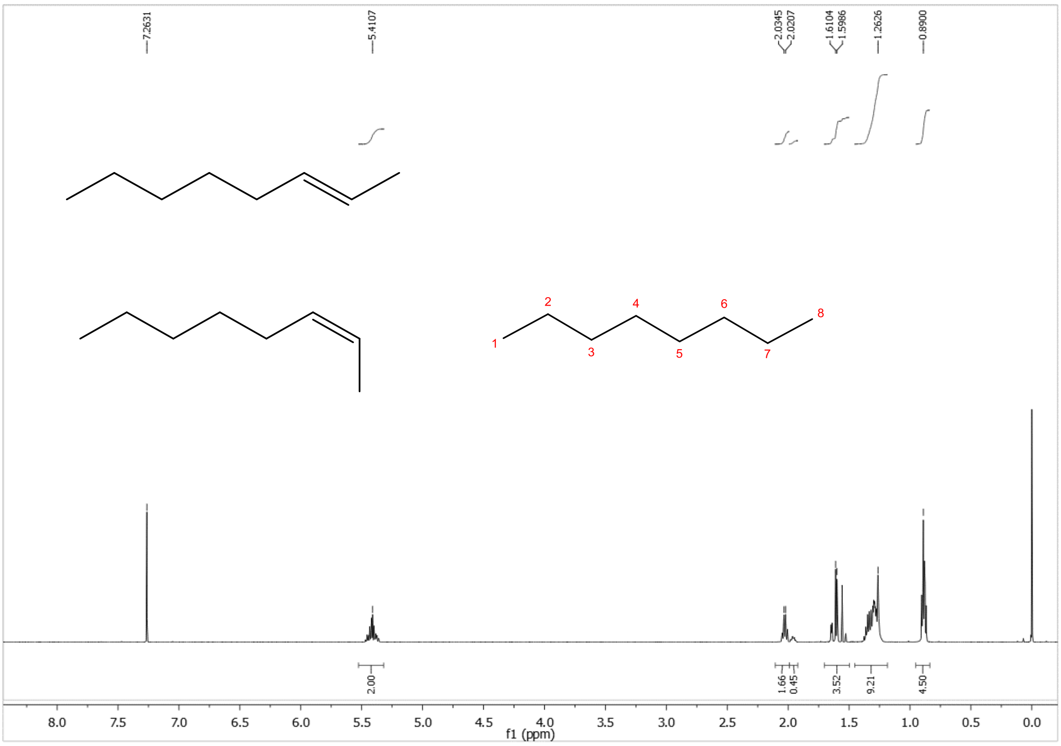

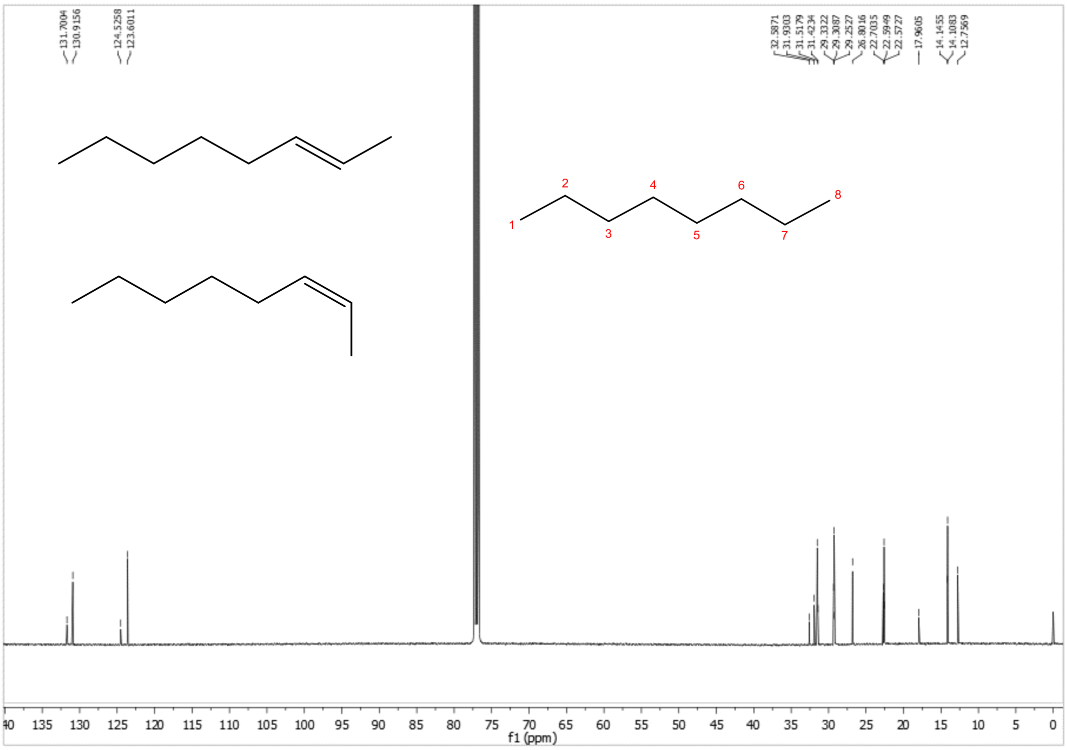

Fig. S29. ^{1}H and ^{13}C NMR (500 MHz, $CDCl_3$) of 2-octene and *n*-octane after the reaction (5 wt% of 2-octene supported on 50 mg of activated carbon, 100 mL 5 wt% ammonia solution, 525 kHz, 30 °C, 6 h, under air).

Yield of *n*-octane determined by GC and ^{1}H NMR were 17% and 20%, respectively.

Methyl oleate reduction

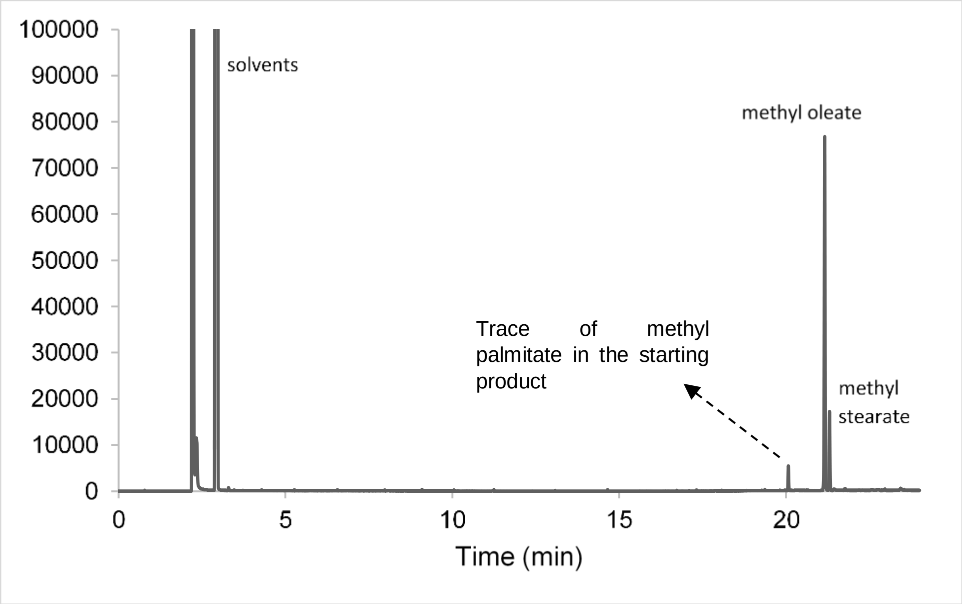

Fig. S30. GC chromatogram of methyl oleate and methyl stearate after the reaction (5 wt% of methyl oleate supported on 50 mg of activated carbon, 100 mL 5 wt% ammonia solution, 525 kHz, 30 °C, 6 h, under air).

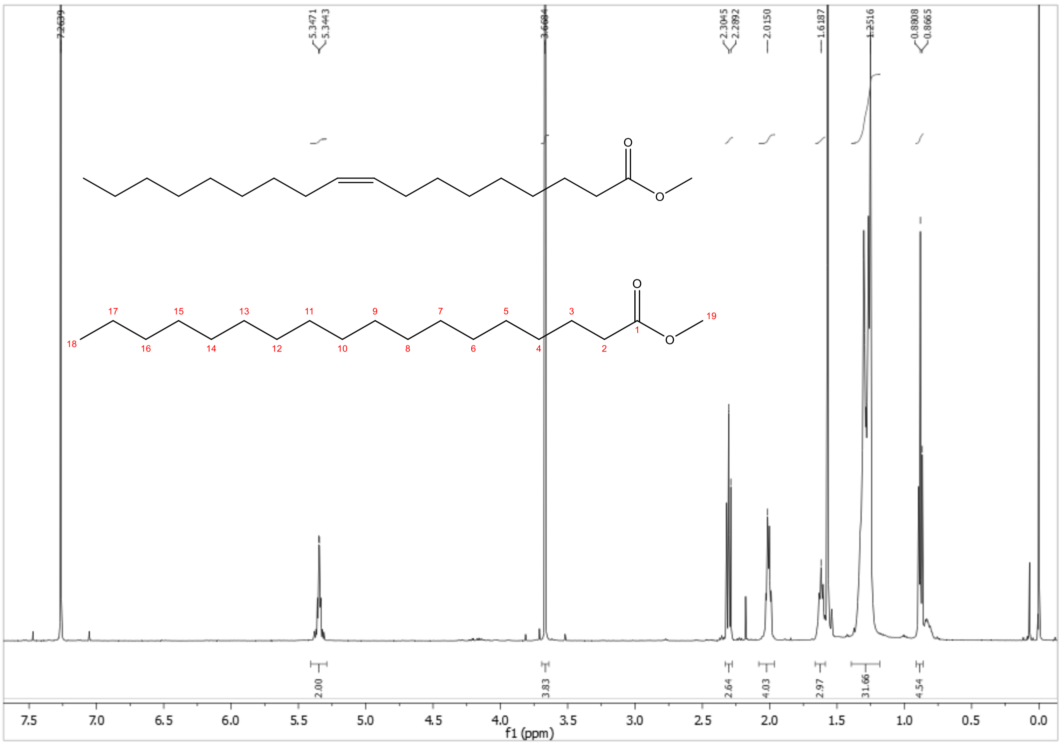

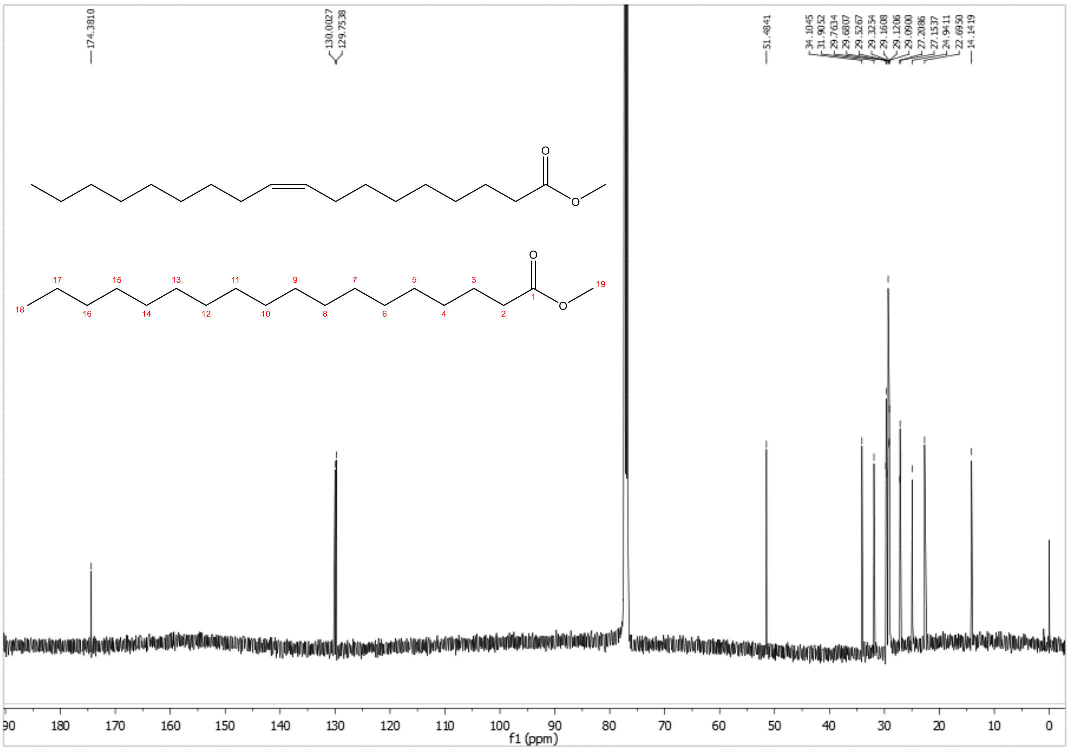

Fig. S31. ^{1}H and ^{13}C NMR (500 MHz, $CDCl_3$) of methyl oleate and methyl stearate after the reaction (5 wt% of methyl oleate supported on 50 mg of activated carbon, 100 mL 5 wt% ammonia solution, 525 kHz, 30 °C, 6 h, under air).

methyl stearate: ^{1}H NMR (500 MHz, $CDCl_3$) δ 3.67 ppm (s, $3H_{19}$), 2.30 ppm (t, $2H_2$, ^{3}J = 8 Hz), 1.62 ppm (m, $2H_3$), 1.25 ppm (m, $28H_{4-17}$), 0.88 ppm (t, $3H_{18}$, ^{3}J = 7 Hz). $^{13}C\{^{1}H\}$ NMR (125 MHz, $CDCl_3$) δ 174.4 (C_1), 51.5 (C_{19}),34.1 (C_2), 31.9 (C_{16}), 29.1-29.7 (C_{4-15}), 24.9 (C_3), 22.7 (C_{17}), 14.1 (C_{18}). HRMS (GC-QTOF) m/z: Calcd for $C_{19}H_{38}O_2$ 298.2872. Found 298.2851

Yield determined by GC and ^{1}H NMR were 18% and 24%, respectively

1-octyne reduction

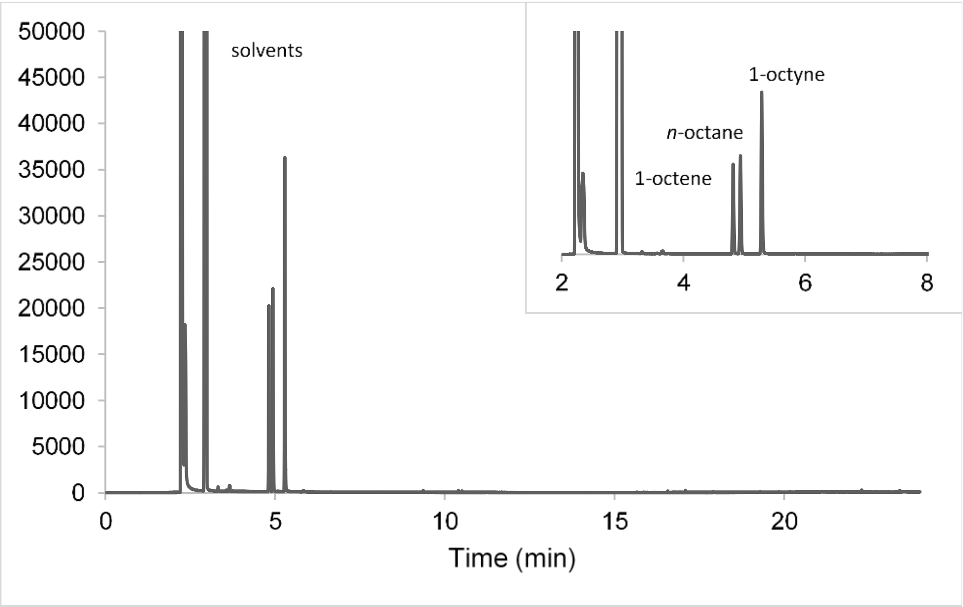

Fig. S32. GC chromatogram of 1-octyne, 1-octene and *n*-octane after the reaction.(5 wt% of 1-octyne supported on 50 mg of activated carbon, 100 mL 5 wt% ammonia solution, 525 kHz, 30 °C, 6 h, under air).

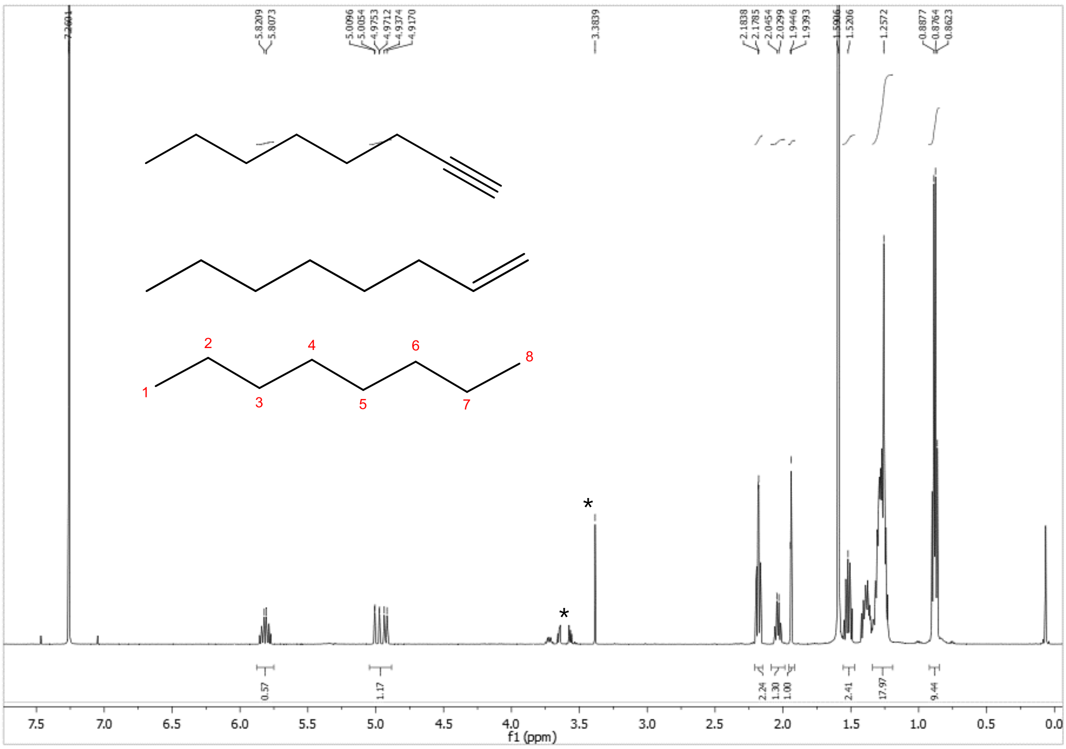

* Pollution of the NMR tube with diglyme

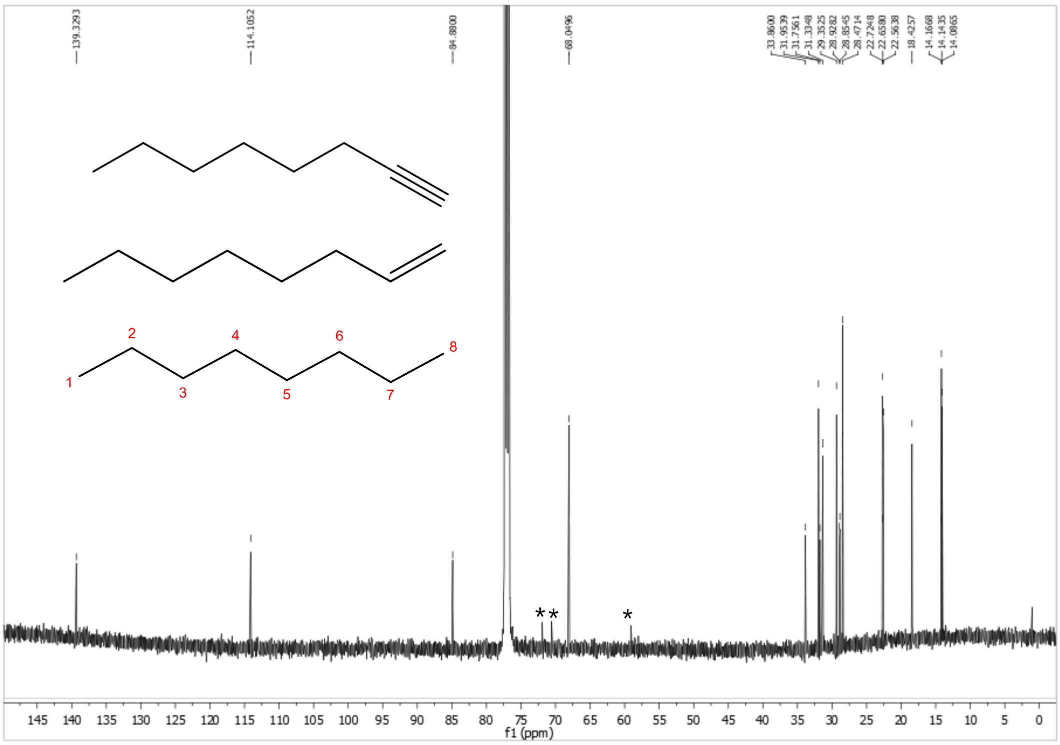

* Pollution of the NMR tube with diglyme

Fig. S33. ^{1}H and ^{13}C NMR (500 MHz, $CDCl_3$) of 1-octyne, 1-octene and *n*-octane after the reaction (5 wt% of 1-octyne supported on 50 mg of activated carbon, 100 mL 5 wt% ammonia solution, 525 kHz, 30 °C, 6 h, under air).

Yield in 1-octene determined by GC and ^{1}H NMR were 26% and 25%, respectively.

Yield in *n*-octane determined by GC and ^{1}H NMR were 29% and 32%, respectively.
